# PDP1 is a key metabolic gatekeeper and modulator of drug resistance in FLT3-ITD-positive acute myeloid leukemia

**DOI:** 10.1038/s41375-023-02041-5

**Published:** 2023-11-07

**Authors:** Islam Alshamleh, Nina Kurrle, Philipp Makowka, Raj Bhayadia, Rahul Kumar, Sebastian Süsser, Marcel Seibert, Damian Ludig, Sebastian Wolf, Sebastian E. Koschade, Karoline Stoschek, Johanna Kreitz, Dominik C. Fuhrmann, Rosa Toenges, Marco Notaro, Federico Comoglio, Jan Jacob Schuringa, Tobias Berg, Bernhard Brüne, Daniela S. Krause, Jan-Henning Klusmann, Thomas Oellerich, Frank Schnütgen, Harald Schwalbe, Hubert Serve

**Affiliations:** 1 Center for Biomolecular Magnetic Resonance (BMRZ), Institute of Organic Chemistry and Chemical Biology, Goethe University Frankfurt, 60438 Frankfurt am Main, Germany; 2 German Cancer Consortium (DKTK), partner site Frankfurt/Mainz, and German Cancer Research Center (DKFZ), Heidelberg, Germany; 3 Department of Medicine, Hematology/Oncology, Goethe University Frankfurt, 60590 Frankfurt, Germany; 4 Frankfurt Cancer Institute, Goethe University Frankfurt, 60596 Frankfurt, Germany; 5 Department of Pediatrics, Goethe University Frankfurt, 60590 Frankfurt, Germany; 6 Georg-Speyer-Haus, Institute for Tumor Biology and Experimental Therapy, 60596 Frankfurt am Main, Germany; 7 Institute of Biochemistry I, Faculty of Medicine, Goethe University Frankfurt, 60590 Frankfurt am Main, Germany; 8 enGene Statistics GmbH, Basel, Switzerland; 9 Department of Experimental Hematology, University Medical Center Groningen, University of Groningen, Groningen, The Netherlands; 10 Centre for Discovery in Cancer Research and Department of Oncology, McMaster University, Hamilton, ON Canada; 11 Project Group Translational Medicine and Pharmacology TMP, Fraunhofer Institute for Molecular Biology and Applied Ecology, 60596 Frankfurt am Main, Germany; 12 Georg-Speyer-Haus; German Cancer Consortium (DKTK), partner site Frankfurt/Mainz, and German Cancer Research Center (DKFZ), Heidelberg, Germany

**Keywords:** Cancer metabolism, Cancer therapeutic resistance

## Abstract

High metabolic flexibility is pivotal for the persistence and therapy resistance of acute myeloid leukemia (AML). In 20–30% of AML patients, activating mutations of FLT3, specifically FLT3-ITD, are key therapeutic targets. Here, we investigated the influence of FLT3-ITD on AML metabolism. Nuclear Magnetic Resonance (NMR) profiling showed enhanced reshuffling of pyruvate towards the tricarboxylic acid (TCA) cycle, suggesting an increased activity of the pyruvate dehydrogenase complex (PDC). Consistently, FLT3-ITD-positive cells expressed high levels of PDP1, an activator of the PDC. Combining endogenous tagging of PDP1 with genome-wide CRISPR screens revealed that FLT3-ITD induces PDP1 expression through the RAS signaling axis. PDP1 knockdown resulted in reduced cellular respiration thereby impairing the proliferation of only FLT3-ITD cells. These cells continued to depend on PDP1, even in hypoxic conditions, and unlike FLT3-ITD-negative cells, they exhibited a rapid, PDP1-dependent revival of their respiratory capacity during reoxygenation. Moreover, we show that PDP1 modifies the response to FLT3 inhibition. Upon incubation with the FLT3 tyrosine kinase inhibitor quizartinib (AC220), PDP1 persisted or was upregulated, resulting in a further shift of glucose/pyruvate metabolism towards the TCA cycle. Overexpression of PDP1 enhanced, while PDP1 depletion diminished AC220 resistance in cell lines and peripheral blasts from an AC220-resistant AML patient in vivo. In conclusion, FLT3-ITD assures the expression of PDP1, a pivotal metabolic regulator that enhances oxidative glucose metabolism and drug resistance. Hence, PDP1 emerges as a potentially targetable vulnerability in the management of AML.

## Introduction

Acute myeloid leukemia (AML) is an aggressive myeloid malignancy with high mortality and poor prognosis particularly in the elderly. Standard therapy of AML consists of intensive cytotoxic therapy, often combined with allogeneic hematopoietic stem cell transplantation. In AML bone marrow, a combination of recurrent and private somatic mutations can be detected in a plethora of genes that normally regulate hematopoietic progenitor proliferation, survival and differentiation [1]. They cause leukemic growth that is characterized by a block in differentiation and deregulated proliferation. One recurrently mutated gene is the Fms-like tyrosine kinase 3 (*FLT3*) which plays a prominent role in hematopoietic stem and progenitor cells and monocyte function [2]. *FLT3* mutations mainly occur as internal tandem duplications (ITDs) of the juxtamembrane domain [3, 4]. They are present in 20-30% of AML patients, are associated with poor prognosis and higher risk of relapse. They have been extensively characterized to be involved in many aspects of AML biology, and to be a therapeutic target, which led to the clinical development of several FLT3 inhibitors for the treatment of FLT3-ITD-positive AML [5–8]. However, as for most AML drugs that specifically target single recurrent mutations, responses are heterogeneous, even among FLT3-ITD-positive patients, and secondary resistance commonly occurs [9, 10].

Besides the presence of activating target mutations, molecular determinants of response and resistance to FLT3 inhibitors are poorly understood. Like most other cancer cells, AML blasts consume high amounts of glucose that is for large parts metabolized into lactate regardless of oxygen levels – a phenomenon known as aerobic glycolysis or the Warburg effect [11–14]. However, enhanced glycolysis towards lactate does not necessarily come at the cost of diminished mitochondrial pyruvate metabolism [15, 16], which is increasingly recognized as an important metabolic feature in cancer in general and specifically in AML [17–24]. Mitochondrial oxidative phosphorylation (OXPHOS) has recently attracted attention, since its activity is associated with AML drug response and resistance to therapies such as cytarabine [25] and venetoclax [26, 27]. Although these dependencies are not fully understood, several metabolic inhibitors are currently being evaluated in preclinical models and clinical trials [28–32]. Understanding the functional connection between oncogenes and the metabolic wiring of AML blasts promises to unravel disease mechanisms with therapeutic relevance.

Modulation of pyruvate dehydrogenase complex (PDC) activity represents a key decision point in the fate of pyruvate, which can either be shuffled into the mitochondria or metabolized into lactate. Pyruvate dehydrogenase phosphatases (PDPs) enhance PDC activity [33], resulting in enhanced shuffling of pyruvate into the mitochondria. Basal PDP1 expression was studied in several human cancers showing entity-specific up or down regulation [34–36]. However, little is known about the regulation of PDP1 by specific oncogenes and its effects on cancer biology. Here, we describe the central role of PDP1 as a regulator of the metabolic fate of pyruvate in FLT3-ITD-positive AML. We show that PDP1 expression is regulated by FLT3-ITD in a context-dependent manner, and that it is centrally involved in the adaptation of AML glucose metabolism to the requirements of proliferation, survival and the therapeutic response to FLT3 inhibition.

## Materials and methods

### Cell culture and reagents

Human AML cell lines were obtained from the DSMZ and were cultured in RPMI 1640 medium (Gibco/ThermoFisher) supplemented with 10% FCS (Sigma-Aldrich), 100 IU/ml penicillin and 100 mg/ml streptomycin (Gibco/ThermoFisher). Molm13 Rho Zero cells were similarly maintained in medium additionally supplemented with 1 mM sodium pyruvate (ThermoFisher) and 50 µg/ml uridine (Sigma-Aldrich). 32D cells were maintained in RPMI 1640 medium containing 10% FCS and 1 ng/ml murine IL3 (Peprotech) [37]. Cells were adapted to FLT3-ITD signaling (IL3 withdrawal) for at least 5 days before performing experiments. For hypoxia experiments, cells were prior adapted to continuous 1% O_2_, 5% CO_2_ in a hypoxia chamber (Biospherix, X3 Xvivo System) for 7 days. Mononuclear cells from peripheral blood or bone marrow of AML patients were purified by Ficoll-Paque™ Premium gradient centrifugation according to the manufacturer’s instructions (GE Healthcare). Sample obtained from the AC220-resistant patient (patient 10) was from peripheral blood aspirate and the patient received prior induction therapy, high-dose cytarabine and AC220. Patient details are listed in Supplementary Table 2. All patients gave informed consent according to the Declaration of Helsinki to participate in the collection of samples. The use of bone marrow aspirates was approved by the Ethics Committee of Frankfurt University Hospital (approval no. SHN-11-2016/SHN-09-2019). The blasts were thawed for experiments and cultivated in X-vivo 10 medium (Lonza) supplemented with 10% HyClone FCS (GE Healthcare), 4 mM L-glutamine (Thermo Fisher), 25 ng/ml hTPO, 50 ng/ml hSCF, 50 ng/ml hFlt3-ligand and 20 ng/ml hIL3 (Peprotech).

### Proliferation, viability and apoptosis assays

Cumulative growth assays were performed at a density of 2.5*10^5^ cells/ml, counted every 48 h and then reseeded to the original density. Competitive growth assays were performed by mixing transduced cells at a ratio of (50:50) with GFP-positive control cells which were monitored by flow cytometry every 48 h. Cellular ATP levels assay was done using CellTiter-Glo (Promega). Apoptosis assays were performed with annexin-APC staining (BD) and analyzed by flow cytometry.

### Seahorse measurement

Cells were suspended in KHB buffer, pH = 7.4, containing 5 mM glucose and 1 mM glutamine at a density of 1-3*10^5^ cells/ml and measured on a XF96 Agilent Seahorse reader.

### RNA extraction, cDNA preparation and qPCR measurement

RNA was extracted using the NucleoSpin RNA kit (Macherey‑Nagel) according to the manufacturer’s instructions and reversely transcribed using SuperScript II (ThermoFisher). Quantitative real-time PCR was performed using SYBR Green JumpStart Taq Ready Mix (Sigma-Aldrich). Primers sequences were (sense: 5′-CTC GTC GGG AAG AAT CGT T-3′, antisense: 5′-GTG GTG GCA GTA ACA TGC AG-3′) for human PDP1 and (sense: 5′-TGC TGA GTG AGG GAA GGA C-3′, antisense: 5′-CAC AGT TAC GGA CGA GAG GA-3′) for mouse PDP1 (Sigma-Aldrich).

### PDP1 knockdown

Lentiviral short hairpin RNA (shRNA) expression vectors were bought as bacterial glycerol stocks from Sigma-Aldrich (shRNA cloned into pLKO.1_puro vector) (sequences in Supplementary Table 3) and plasmid DNA was prepared with NucleoSpin DNA kit (Macherey‑Nagel). For the in vivo AC220 sensitivity experiments with MV4-11 cells (AC220 and PDP1 combination in vivo), the same construct backbones were used but with a replacement of puromycin resistance cassette with a fluorescence expression cassette (GFP or E2Crimson). PDP1 siRNAs (Sigma-Aldrich) (sense: 5′-CGG UAU UUG AGG AUC AGA A-3′, antisense: 5′-UUC UGA UCC UCA AAU ACC G-3′) and scrambled negative control siRNA duplex (Origene) were transfected with Viromere (Biozyme lypocalyx) reagent at a concentration of 100 nM.

### PDP1 overexpression

Coding sequence of human PDP1 cDNA was amplified from RPMI 8226 cells via a standard PCR reaction using the primers: sense: 5′-GGG GGG GAT CCA CCG GTC GCC ACC ATG CCA GCA CCA ACT CAA CTG-3′; antisense: 5′-GGG CGT CGC GAC TCA TTC TTG GTT TTG ATA CGC CCC-3′. The PCR product was cloned into an overexpression (OE) vector (SIHW, with hygromycin selection cassette, modified from pHR’SINcPPT-SBW Ward et al., 2011 [38]) via BamHI and NruI restriction enzymes.

### Generation of Rho Zero cells

Molm13 Rho Zero were generated by introducing mutations into the mitochondrial polymerase gamma (POLG) by lentiviral transduction of Cas9-positive Molm13 cells using the CRISPR/Cas9 system and subsequent subcloning under supplementation with 1 mM sodium pyruvate (ThermoFisher) and 50 µg/ml uridine (Sigma-Aldrich). Guide RNA (sgRNA sense: 5′-CAC CGc tgc acc agg aat acc tga-3′, antisense: 5′-AAA Ctc agg tat tcc tgg tgc agC-3′) was cloned into a pLentiCRISPRv2 ΔCAS9-BFP vector by target-specific oligonucleotide cloning following the GoldenGate protocol [39].

### Xenotransplantation experiments

Ten- to fourteen-week-old, non-irradiated NOD SCID with interleukin-2 receptor knockout (NSG) mice were transplanted intravenously with 1*10^5^ Molm13 or 5*10^5^ MV4-11 cells transduced with either NTC or shPDP1 shRNA. For the in vivo competition assay, MV4-11 cells were transduced with either pLKO.1-EGFP shNTC or pLKO.1-EGFP shPDP1. GFP-positive cells were mixed in a 1:1 ratio with MV4-11 cells transduced with pLKO.1-E2Crimson shNTC prior to transplantation [40]. AC220 treatment was initiated at day 29 (when engraftment was first detected (1%)) and the treatment regimen was as described previously [41]. All animal studies were approved by the local German government (Regierungspräsidium Darmstadt) in Hesse, Germany (FK/1117 for the overall survival transplantation and FK/2044 for the in vivo competition assay).

### Extraction of metabolites and NMR sample preparation and measurement

Metabolites were extracted using methanol/chloroform-based protocol. 10 million cells were extracted and the polar layer was dried and resuspended in NMR phosphate buffer. Samples were measured on a Bruker 600 MHz AVIIIHD spectrometer equipped with a nitrogen cooled triple resonance probe head (TCI Prodigy) at 25 °C, TD: 32 K, 512 scans using noesygppr1d pulse program. Metabolites identification and quantification was performed using Chenomx, Metabolab [42] and Sparky (UCSF) [43] as described previously [44].

### In silico analysis of the TCGA LAML database for PDP1 mRNA expression

HG38-aligned gene-level RNAseq read counts (HTSeq), sample and clinical data for the TCGA LAML cohort were downloaded from the TCGA data portal. Differential gene expression analysis was done using edgeR 3.30.3 [45] and limma 3.44.3 [46]. A between-sample normalization factor was calculated using trimmed mean of M-values (TMM). Library size-adjusted read counts were voom-transformed and linear models comparing FLT3-ITD vs FLT3-WT patient samples were fit to the expression values using limma.

### PDP1 endogenous tagging

Endogenous tagging was performed a previously described (Thöne et al., 2019). Briefly, a DNA double strand break was introduced at the C-terminus of the PDP1 gene locus in Cas9 -positive 32D/FLT3-ITD cells using the sgRNA expression vector pLentiCRISPR ΔCas9-E2Crimson equipped with a PDP1 gene-specific sgRNA constructed from the annealed oligonucleotides 5′-CAC CGg tag ggg cat acc aaa acc-3′ (sense) and 5′-AAA Cgg ttt tgg tat gcc cct acC-3′ (antisense). Simultaneously with this vector, a generic tagging vector (pGTag Hygro EGFP cLAP -1 (available at Addgene #194312)) was co-electroporated from which the desired tag was liberated in situ by CRISPR/Cas9. Using the cell’s own non-homologous end-joining (NHEJ) repair pathway, the tag was now inserted into the cleaved gene locus and correctly integrated cell clones were selected with 400 µg/ml Hygromycin via the inserted hygromycin resistance cassette. Electroporation was performed using the Neon transfection system (ThermoFisher) (1200 volt, 3 pulses with 20 ms pulse width) electroporating 2*10^6^ Cas9^+^ FLT3-ITD 32D cells with both vectors (10 µg each). After subcloning, gene to tag and tag to gene junctions of individual clones were amplified by PCR and Sanger-sequenced to confirm correct integration of the tag. Flow cytometry was performed to analyze GFP signal and Western blot was performed to validate the correct fusion protein size.

### Genome wide CRISPR/Cas9 screen to identify PDP1 regulatory genes

Mouse Brie library vector pool (80.000 gRNAs) (Addgene #73633) [47] after receipt from the vendor was transformed (400 ng) into electrocompetent E. coli (STBL4 (ThermoFisher)), plated onto LB-agar bioassay plates, further expanded in liquid LB medium and purified according to manufacturer’s instructions (Zymo Research Midi/Maxi-prep kit). Lentiviral supernatants were generated by cotransfection with pMD2.G and psPAX2 lentiviral packaging vectors into 293 T lentiX cells (Takara). 2.5*10^8^ PDP1-tagged Cas9 + 32D cells were transduced with the library virus at an MOI of 20% (to guarantee 500-fold representation of each gRNA and individual transductions) (virus production is described in the supplementary). 24 h later, cells were selected with puromycin (2 µg/ml) for 48 h and another 72 h later, 5*10^7^ cells were FACS sorted on a BDFACS ARIAIII Cell sorter (BD Biosciences) into 3 subpopulations according to their GFP intensity (2.5*10^6^ cells with the lowest 5% GFP, 45*10^6^ cells with intermediate GFP intensity (90% of the cells) and 2.5*10^6^ cells with the highest 5% GFP). DNA was then extracted, sgRNA integrants were amplified and sequenced by Illumina next generation sequencing.

### Bioinformatics analysis

We used MAGeCK MLE ([48, 49]) to call differentially represented sgRNAs in each sample compared to the intermediate baseline (the intermediate GFP population). The algorithm model counts as a negative binomial and employs a maximum likelihood estimation (MLE) approach to compute a β score for each gene. A positive β score indicates positive selection, whereas a negative β score indicates negative selection typical of essential genes.

### Statistical analysis

Statistical analysis was performed with confidence interval of 95%. IC50 determination was performed by nonlinear regression. Experiments are plotted as mean ± standard deviation (SD) for *n* = technical replicates. Each experiment was performed with biological duplicates or triplicates.

## Results

### FLT3-ITD shifts glucose metabolism towards OXPHOS

**Fig. 1:**
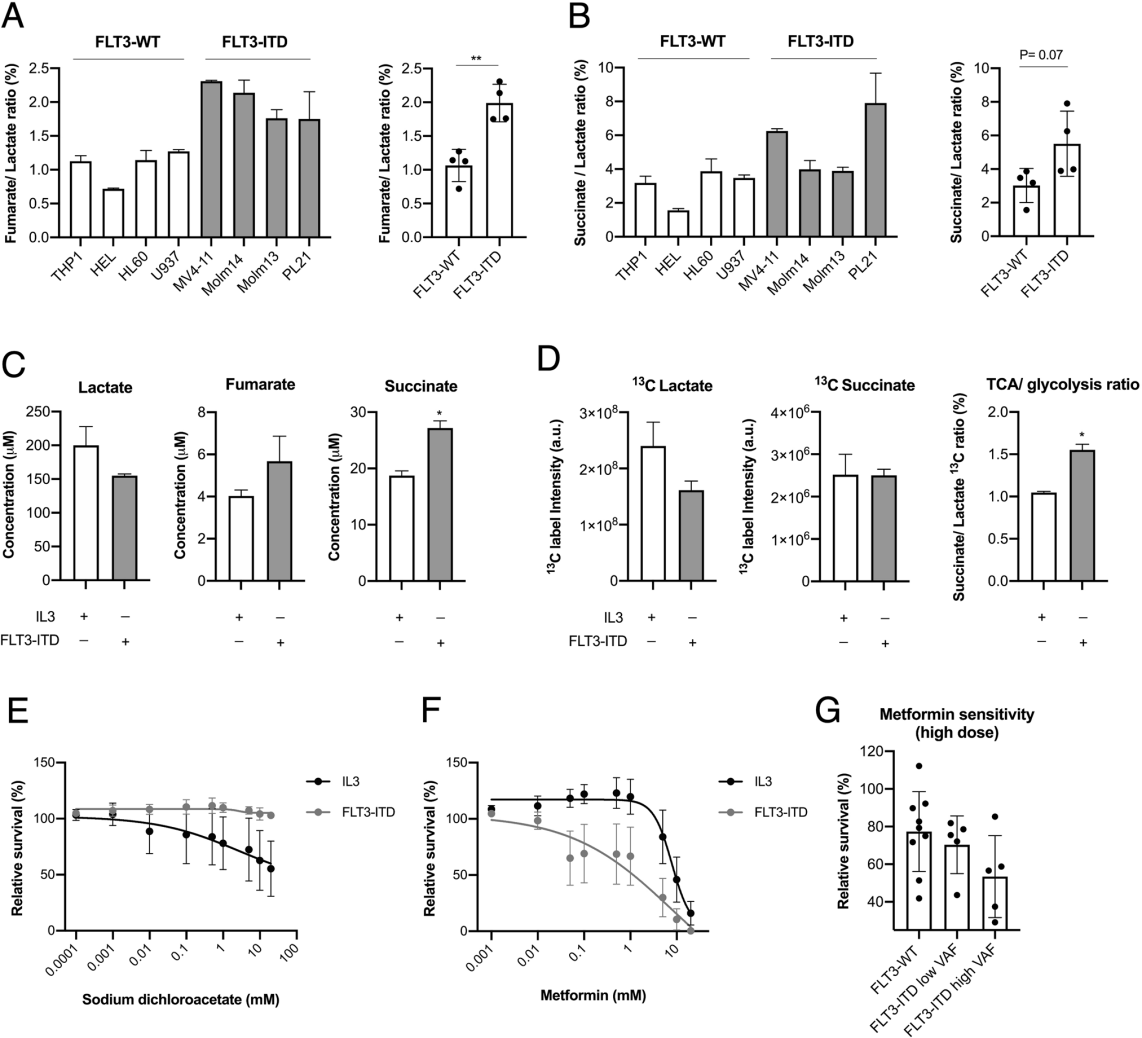
FLT3-ITD cells display high OXPHOS metabolism and are sensitive to mitochondrial inhibition. Representative NMR measurements of fumarate/lactate ratio (**A**) and succinate/lactate ratio (**B**) (TCA/glycolysis) in FLT3-ITD compared to FLT3-WT AML cell lines (*n* = 2 biological replicates). **C** Representative NMR measurements of lactate, succinate and fumarate levels in 32D cells under the influence of FLT3-ITD kinase activity in comparison to IL3 stimulation (24 h stimulation) (*n* = biological replicates). **D** NMR measurements of ^13^C label incorporation into succinate and lactate (together with their ratio) after 24 h of labelling with ^13^C glucose (*n* = 2 technical replicates). **E** Cellular sensitivity to sodium dichloroacetate in 32D cells under IL3 compared to FLT3-ITD signaling (treated for 72 h) (*n* = 3). **F** IC_50_ curve of metformin sensitivity in 32D cells under IL3 compared to FLT3-ITD signaling (treated for 72 h) (*n* = 5). **G** Metformin sensitivity in FLT3-ITD-positive *vs* FLT3-WT primary AML blasts (5 mM for 72 h) (*n* = 10 and 9, respectively). Data are presented as mean values ± SD. In (**B**) and (**C**), unpaired Student’s *t*-test was performed (**P ≤ 0.01, *P ≤ 0.05).

To characterize the effects of FLT3-ITD on glucose metabolism, we first analyzed a panel of eight different AML cell lines (THP1, HEL, HL60, U937, MV4-11, Molm13, Molm14, PL21) by nuclear magnetic resonance (NMR) spectroscopy to assess their mitochondrial TCA cycle and their glycolytic activity, taking the ratio of the concentrations of the tricarboxylic acid (TCA) cycle intermediates fumarate to lactate (Fig. 1A) and succinate to lactate as indicators (Fig. 1B). We observed a 2-fold higher TCA/glycolysis ratio in FLT3-ITD-positive compared to FLT3-ITD-negative AML cell lines. In addition, we performed Seahorse measurements of the mitochondrial respiration capacity in those cell lines and took the ratio between the oxygen consumption rates (OCR) and the extracellular acidification rates (ECAR) as an indicator of the cellular metabolic status. In agreement with the NMR data, we observed higher OCR/ ECAR ratios in the FLT3-ITD-positive AML cell lines (Supplementary Fig. 1A). To establish a causal relationship between FLT3-ITD and increased TCA cycle activity, we transduced the murine cytokine-dependent hematopoietic progenitor cell line 32D with an FLT3-ITD expression construct [37]. This model allows to directly compare the specific effects of FLT3-ITD with default cytokine signaling (IL3) in one syngeneic cell model [37, 50]. Here, cells growing without IL3, but under the control of FLT3-ITD, showed significantly higher succinate and fumarate levels whereas lactate levels were lower, when compared to IL3-dependently growing 32D cells (in which FLT3-ITD kinase activity was abrogated by incubation with 5 nM of the tyrosine kinase inhibitor AC220) (Fig. 1C). From these experiments, we concluded that the FLT3-ITD oncogene induced higher TCA cycle activity.

Next, we explored whether this increase in TCA cycle activity was fueled by enhanced glycolysis. We performed a tracer-based NMR assay using uniformly labelled ^13^C glucose to analyze the dynamic flux of pyruvate at the interphase between glycolysis and the TCA cycle. Total label incorporation into lactate was reduced under FLT3-ITD signaling when compared to IL3 stimulation, while label incorporation into succinate was conserved. Hence, the succinate/lactate ratio was increased under the influence of FLT3-ITD (Fig. 1D), suggesting that in the presence of FLT3-ITD, a higher proportion of pyruvate is translocated into the mitochondria to fuel the TCA cycle, at the expense of being metabolized to lactate. Together, these data show that FLT3-ITD triggers a metabolic profile that is characterized by a dominant oxidative glucose metabolism.

To study the dependence of FLT3-ITD positive cells on mitochondrial energy production, we assessed the effects of various glycolysis and mitochondrial metabolism inhibitors. First, we demonstrated that 32D cells are significantly less sensitive to glycolysis inhibition by sodium dichloroacetate (PDHK1 inhibitor) when under the influence of FLT3-ITD compared to IL3 signaling (Fig. 1E). In contrast, FLT3-ITD signaling renders the cells significantly more sensitive to metformin (Fig. 1F), which inhibits OXPHOS at high doses [51]. Similarly, we observed higher sensitivity to other inhibitors of the electron transport chain (ETC) (atpenin A5 (complex II inhibitor) and antimycin A (complex II inhibitor)) in cells growing with FLT3-ITD kinase activity (Supplementary Fig. 1B–D). This observation was also confirmed in primary patient-derived AML blasts ex vivo, where FLT3-ITD-positive blasts were more sensitive to metformin (Fig. 1G). Interestingly, this correlated with FLT3-ITD variant allele frequency (VAF) where samples with higher VAF showed more sensitivity. These findings indicate that FLT3-ITD-positive cells are more dependent on mitochondrial energy production than FLT3-ITD-negative cells.

### FLT3-ITD cells express high levels of PDP1

**Fig. 2:**
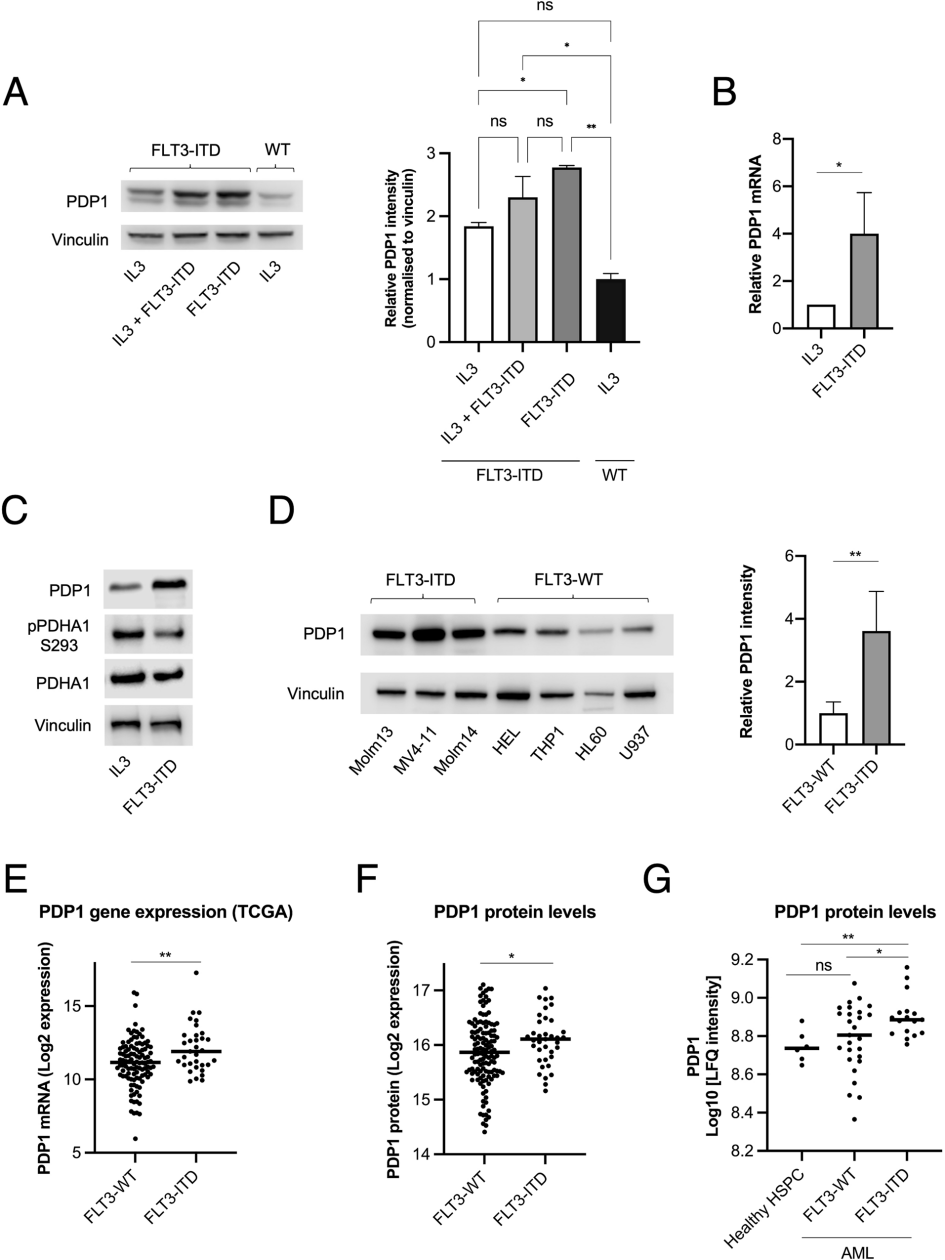
FLT3-ITD cells express high PDP1 levels. **A** PDP1 protein levels in 32D cells under FLT3-ITD kinase activity, IL3 signaling or their combination (*n* = 2). **B** qPCR measurements of PDP1 mRNA levels in 32D cells under FLT3-ITD vs IL3 signaling (*n* = 4). **C** Western blot of S293 inhibitory phosphorylation levels of PDHA1 (*n* = 3). **D** PDP1 protein levels in FLT3-ITD-positive AML cell lines compared to their FLT3-WT counterparts (*n* = 2). **E** PDP1 mRNA expression levels in primary AML blasts (FLT3-WT vs FLT3-ITD, *n* = 108 and 33, respectively) extracted from the TCGA AML cohort (https://tcga-data.nci.nih.gov/tcga). **F** PDP1 protein levels in AML blasts (FLT3-WT vs FLT3-ITD) (*n* = 37 and 128, respectively) extracted from the AML proteogenomic cohort [52] (https://www.cell.com/cancer-cell/fulltext/S1535-6108(22)00058-7). **G** PDP1 protein levels in primary AML CD34^+^ cells and healthy CD34^+^ HSPCs (Healthy HSPCs vs FLT3-WT vs FLT3-ITD, *n* = 6, 17 and 27, respectively) extracted from Boer et al. 2018 [53] (https://www.cell.com/cancer-cell/fulltext/S1535-6108(18)30374-X). Data are presented as mean values ± SD. In (**D**), (**E**) and (**F**), unpaired Student’s *t*-test was performed (**P ≤ 0.01, *P ≤ 0.05). In (**A**) and (**G**), one-way ANOVA with Bonferroni’s multiple comparisons was performed (**P ≤ 0.01, *P ≤ 0.05).

Having shown that FLT3-ITD causes a switch in glucose metabolism in favor of the TCA cycle, we sought to characterize the effects of FLT3-ITD on the pyruvate dehydrogenase complex (PDC) because it is the key regulator of pyruvate metabolism. In 32D cells, we observed significantly higher PDP1 protein and mRNA levels in cells that grew under FLT3-ITD signaling in comparison to those growing under stimulation with IL3 (Fig. 2A, B), and this elevation was maintained even under mixed FLT3-ITD and IL3 signaling (Fig. 2A). These higher PDP1 levels were associated with lower S293 inhibitory phosphorylation of PDHA1 (Fig. 2C). We confirmed this finding in a panel of human AML cell lines, which consistently showed higher PDP1 levels in FLT3-ITD cells compared to FLT3-WT cells (Fig. 2D). Additionally, we performed an in silico analysis on the TCGA LAML cohort (141 AML patients with FLT3-WT or FLT3-ITD). Here, we analyzed the data for PDP1 expression in these patients, 33 of whom were FLT3-ITD-positive. Consistent with the findings in cell lines, the occurrence of FLT3-ITD mutations significantly correlated with higher PDP1 mRNA levels in this cohort (Fig. 2E). Moreover, we performed another in silico analysis of PDP1 protein levels in an AML proteogenomic cohort [52] (FLT3-WT *vs* FLT3-ITD, *n* = 37 and 128, respectively) where we also observed higher PDP1 protein levels in FLT3-ITD AML cells compared to FLT3-WTs (Fig. 2F). The same results were also replicated from another proteomic cohort [53] where PDP1 protein was higher in the leukemic stem/progenitor cell-enriched CD34^+^ compartment of FLT3-ITD compared to FLT3-WT AML samples, and this difference was even more significant when compared to healthy CD34^+^ hematopoietic progenitor cells (HSPCs) (Fig. 2G).

### RAS signaling regulates PDP1 expression

**Fig. 3:**
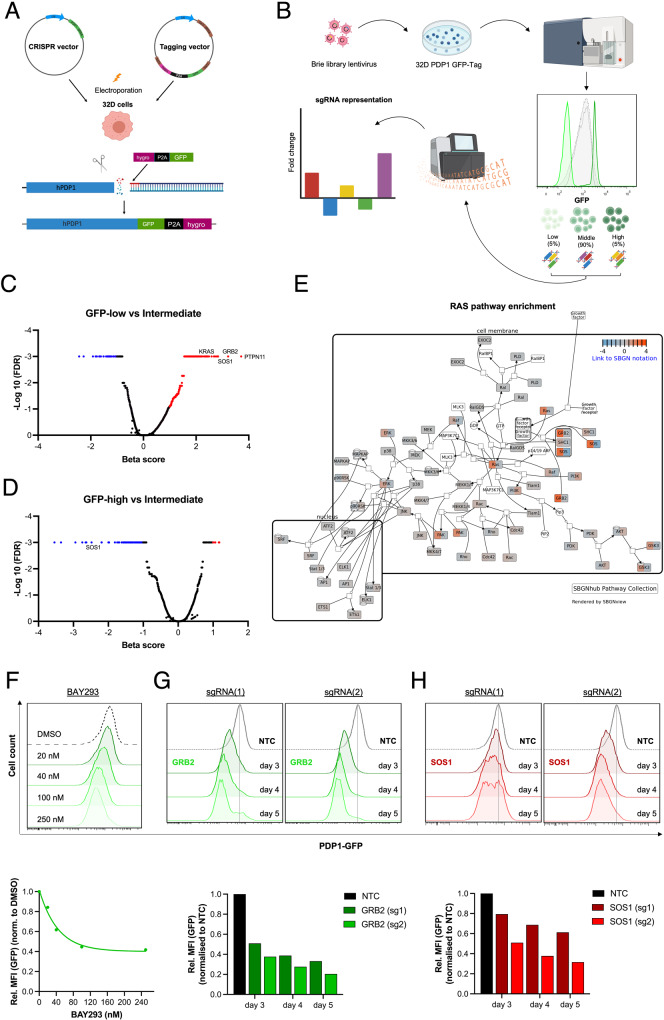
Endogenous tagging of PDP1 reveals the role of RAS signaling in mediating FLT3-ITD-induction of PDP1 expression. **A** General strategy of PDP1 tagging using a CRISPR vector to induce a double strand break at the C-terminus of the gene locus and a generic tagging vector to incorporate a GFP gene sequence. **B** Experimental design of the CRISPR screen and FACS sorting of the cells into 3 subpopulations according to their GFP florescence intensity (lowest 5%, intermediate 90% and highest 5%). Parts of the figure were designed using BioRender. **C** Volcano plots of significantly enriched genes in the GFP-low population compared to the middle 90%, and (**D**) of the significantly depleted genes in the GFP-high cells compared to the intermediate 90% population. **E** Graphical illustration of the RAS pathway genes and their enrichment scores in the GFP-low cells. **F** Flowcytometry analysis of PDP1 levels in PDP1-tagged 32D FLT3-ITD cells upon treatment with the KRAS-SOS inhibitor (BAY-293). **G** PDP1 levels in PDP1-tagged 32D FLT3-ITD cells after GRB2 or (**H**). SOS1 knockouts (measured by flowcytometry of PDP1-GFP tag). Quantitative mean florescence intensity (MFI) values are also shown.

Next, we were interested to understand the FLT3-ITD-dependent signaling events leading to the induction of PDP1 expression. We endogenously tagged PDP1 in 32D FLT3-ITD cells with a GFP cDNA at the 3’ end of the coding region in its gene locus (Fig. 3A). These cells expressed one of the two PDP1 alleles as a PDP1-GFP fusion protein (two protein sizes are observed (56 kDa (wild type protein) and 93 kDa (fusion protein) (Supplementary Fig. 2A) which allows to monitor PDP1 expression via flow cytometry. These cells had comparable growth behavior to their WT counterparts (Supplementary Fig. 2B) and their PDP1 expression (assessed by GFP intensity) was equally responsive to IL3 withdrawal (elevated when cells were grown under FLT3-ITD signaling) (Supplementary Fig. 2C). Sequencing confirmed correct tag integration without any base pair deletions or insertion (Supplementary Fig. 2D).

We subsequently used these tagged cells to perform a genome-wide CRISPR screen, where we transduced them with the murine Brie library [47], and then FACS-sorted them according to their GFP florescence intensity (lowest 5%, intermediate 90% and highest 5%) (Fig. 3B). These three subpopulations were then sequenced and the relative distribution of all sgRNAs was analyzed in a comparative manner. We anticipated to find enrichment for sgRNAs that target genes which induce PDP1 expression within the lowest 5% population (PDP1 levels drop and hence GFP intensity goes down when abrogating its inducers) while genes that suppress PDP1 expression were anticipated to be enriched in the highest 5% population.

Interestingly, genes that are involved in the RAS signaling pathway, namely SOS1, GRB2, SHOC2 and PTPN11 scored amongst the top enriched genes in the GFP-low population (PDP1 inducers) (Fig. 3C), while they were depleted from the GFP-high population (PDP1 repressors) (Fig. 3D, E), suggesting a substantial role of the RAS signaling pathway in regulating PDP1 expression. To confirm this, we pharmacologically inhibited RAS signaling by treating the 32D FLT3-ITD PDP1-tagged cells with BAY-293, a selective inhibitor of the KRAS-SOS1 interaction. Indeed, we observed a strong reduction in their PDP1 expression in a dose-dependent manner (Fig. 3F). We further validated this by the genetically depleting GRB2 and SOS1 individually in those cells (CRISPR knockout). Similarly, we observed a decline in PDP1 signal over time (Fig. 3G, H).

### PDP1 is a targetable metabolic dependency in FLT3-ITD-positive AML

**Fig. 4:**
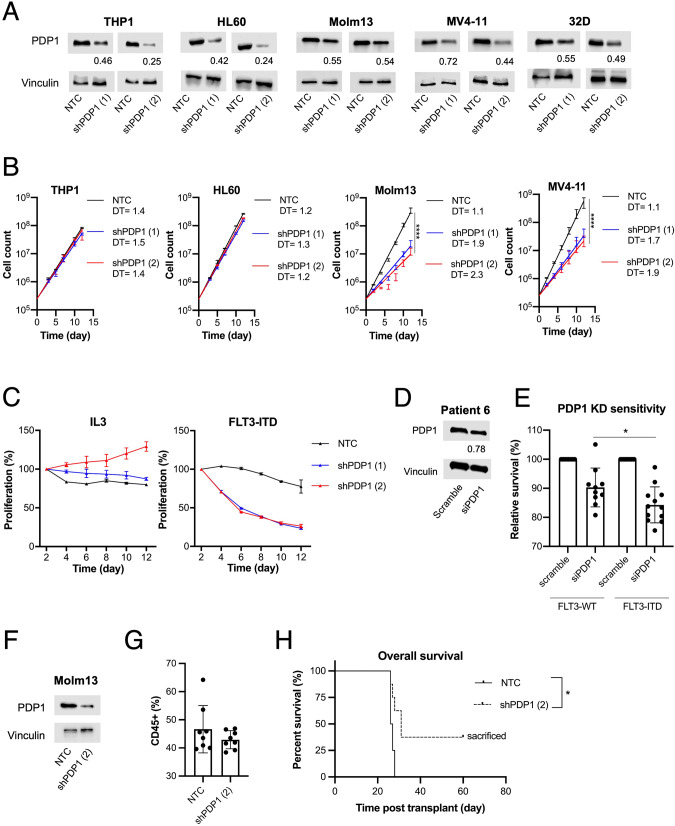
PDP1 targeting exclusively abrogates FLT3-ITD-positive AML cells. **A** Western blot of PDP1 knockdown using PDP1-shRNA in AML cell lines and 32D cells. **B** Cumulative growth assays upon PDP1 knockdown in FLT3-WT vs FLT3-ITD cell lines (*n* = 2). Data are presented as mean values ± SD. Two-way ANOVA with Bonferroni’s two group comparisons was performed (****p ≤ 0.0001, *p ≤ 0.05). **C** Competitive growth assay upon PDP1 knockdown in 32D cells signaling with either IL3 (left) or FLT3-ITD (right) (*n* = 2). **D** Representative Western blot of PDP1 knockdown using PDP1-siRNA in primary AML blasts (patient 6). **E** Impact of PDP1 knockdown on FLT3-ITD-positive compared to FLT3-ITD-negative primary AML blasts from different patients (*n* = 12 and 10, respectively). **F** Western blot of PDP1 knockdown (shPDP1 [2]) in Molm13 cells prior to their transplantation into NSG mice. **G** Human CD45+ ratio in the peripheral blood of the mice measured at day 20 post-transplantation. **H** Overall survival of NSG mice transplanted with either control or PDP1-knockdown Molm13 cells (*n* = 8 per group) (1*10^5^ cell per mouse). Three mice in the PDP1-knockdown group did not succumb to leukemia and were sacrificed on day 60. Data are presented as mean values ± SD. In (**E**), unpaired Student’s *t*-test was performed (*P ≤ 0.05). In (**H**), Gehan-Breslow-Wilcoxon test was performed (*P ≤ 0.015).

Next, we assessed the functional relevance of PDP1 expression in FLT3-ITD-positive AML. We performed PDP1 knockdown in FLT3-ITD (Molm13 and MV4-11) and FLT3-WT (THP1 and HL60) cell lines using an shRNA approach (Fig. 4A). We observed a pronounced growth disadvantage in FLT3-ITD cells after PDP1 knockdown while FLT3-WT cells were mostly unaffected (Fig. 4B). The growth disadvantage correlated with the knockdown efficiency. The 32D FLT3-ITD model allowed to directly compare the functional relevance of PDP1 expression under IL3 signaling *vs* oncogenic FLT3-ITD signaling. We performed a competitive growth assay between PDP1-knockdown cells and GFP-positive control cells (mixed in 50:50 ratio) and tracked the proportion of GFP-positive cells as an indicator of proliferative divergence. We observed a growth disadvantage upon PDP1 knockdown when the cells were under the influence of FLT3-ITD signaling (Fig. 4C, right). In contrast, PDP1 knockdown did not confer a growth disadvantage when the cells grew with IL3 (Fig. 4C, left). We confirmed these findings in a set of primary patient-derived AML blasts using an siRNA approach. The survival of bone marrow- or peripheral blood-derived blasts from AML patients was assessed in short-term cultures upon small interfering RNA (siRNA)-mediated PDP1 knockdown and compared with non-targeting (scrambled) siRNA controls. Although only 22% knockdown efficiency was achieved (Fig. 4D), a significantly higher sensitivity to PDP1 knockdown was observed in the FLT3-ITD-positive compared to FLT3-WT AML blasts from different patients (Fig. 4E). To functionally assess the role of PDP1 in leukemia development in vivo, we transplanted (FLT3-ITD-positive) Molm13 cells transduced with either non-targeting control (NTC) or PDP1 shRNA expression vectors (Fig. 4F) into immunocompromised NOD/SCID interleukin-2 receptor gamma knockout (NSG) mice. All mice showed engraftment on day 20 post-transplant, as assessed by hCD45-positive cells in peripheral blood (>40%) (Fig. 4G). Consistent with the in vitro data, PDP1 knockdown delayed disease development and prolonged the overall survival significantly (Fig. 4H). Three out of eight mice in the PDP1 knockdown group did not succumb to leukemia and were sacrificed on day 60.

### PDP1 abrogation leads to growth disadvantage by causing mitochondrial insufficiency

**Fig. 5:**
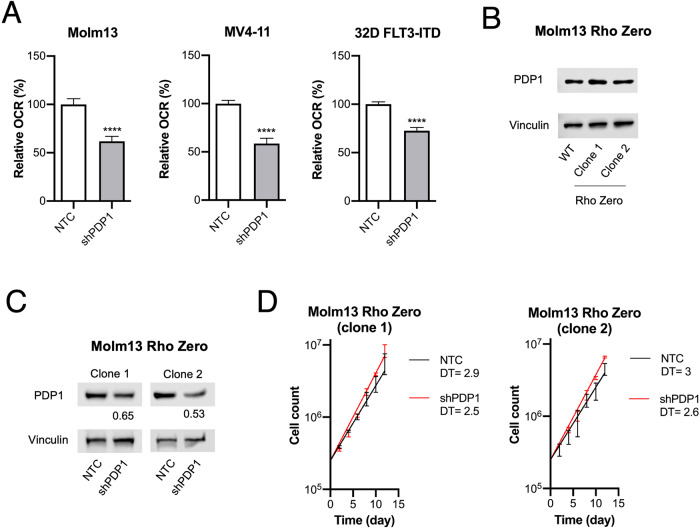
PDP1 exerts its role by its metabolic functions. **A** Representative oxygen consumption rate (OCR) measurements upon PDP1 knockdown in 32D FLT3-ITD (shPDP1 [1]), Molm13 and MV4-11 cells (shPDP1 [2]). The experiment was replicated twice. **B** PDP1 levels in Molm13 Rho Zero cells. **C** Western blot of PDP1 knockdown in Molm13 Rho Zero cells (shPDP1 [2]). **D** Cumulative growth assay of Molm13 Rho Zero upon PDP1 knockdown. Data are presented as mean values ± SD. In (**A**), unpaired Student’s *t*-test was performed ****p ≤ 0.0001, **p ≤ 0.01, *p ≤ 0.05).

So far, our experiments showed that FLT3-ITD-dependent RAS activation triggers a metabolic switch towards more dominant oxidative glucose metabolism, and that at the same time FLT3-ITD is associated with high PDP1 expression relevant for proliferation and survival. Next, we sought to establish a functional relationship between those two findings and to confirm that PDP1-dependency is indeed due to its metabolic function. First, we confirmed that PDP1 knockdown in FLT3-ITD-driven cells resulted in decreased mitochondrial metabolism, assessed by a reduced OCR (Fig. 5A), suggesting that PDP1 in these cells indeed relays the FLT3-ITD-mediated metabolic switch. On the other hand, we observed a significant increase in OCR upon PDP1 overexpression in Molm13 cells (Supplementary Fig. 3A). In order to show that PDP1 dependency is indeed due to its metabolic function, we made use of another model, where we engineered an FLT3-ITD-expressing leukemia cell line that proliferates independently of mitochondrial energy production (Rho Zero cells). Here, we abrogated the mitochondrial polymerase γ (POLG) in Molm13 cells (Supplementary Fig. 3B), which resulted in the termination of mitochondrial DNA replication (Supplementary Fig. 3C), and thus in an impaired ETC. These cells have abolished respiration capacity (Supplementary Fig. 3D), and instead exert high glycolytic activity (Supplementary Fig. 3E). Interestingly, they maintained high PDP1 levels despite their lack of ETC activity (Fig. 5B). Consistent with our hypothesis, PDP1 knockdown did not impact the proliferation capacity of these cells (Fig. 5C, D).

### PDP1 is essential for FLT3-ITD cells under hypoxia and allows for a rapid OXPHOS metabolic switch upon oxygen availability

**Fig. 6:**
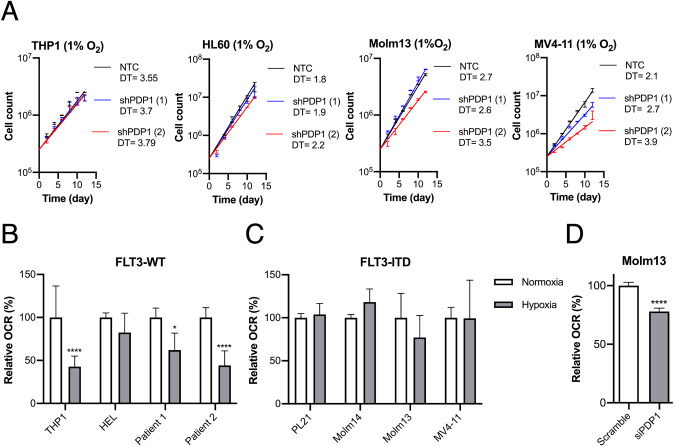
PDP1 is essential for FLT3-ITD cells under hypoxia and mediates their metabolic flexibility. **A** Cumulative growth assays upon PDP1 knockdown under hypoxic conditions in FLT3-WT compared to FLT3-ITD cell lines. **B** Seahorse OCR measurements of respiration capacity in FLT3-WT AML cell lines and primary patient-derived AML blasts after adaption to hypoxic conditions (*n* = 2). **C** Respiration capacity of FLT3-ITD cells after adaption to hypoxia (representative experiment with two biological replicates). **D** Respiration capacity of Molm13 cells adapted to hypoxia (7 days) with prior PDP1 knockdown (3 days before the experiment). Statistical analysis for this experiment is shown for five technical replicates. Data are presented as mean values ± SD. In (**B**), two-way ANOVA with Bonferroni’s two group comparisons was performed (****p ≤ 0.0001, *p ≤ 0.05).

Having established the prominent role of PDP1 in FLT3-ITD-regulated glucose metabolism under normoxic conditions (21% O_2_), we investigated its role in adjusting leukemic cells to changing oxygen availability. Therefore, we adapted AML cell lines for at least 7 days to chronic hypoxia prior to performing growth assays. Adapted cells were used to perform cumulative growth assays under hypoxic conditions and demonstrating that PDP1 knockdown caused a similar growth disadvantage in FLT3-ITD-positive AML cells as under normoxic conditions (Fig. 6A). In contrast, knockdown of PDP1 did not change the growth properties of FLT3-WT cells. We were furthermore interested in how chronic hypoxia influenced the cellular capacity to immediately start oxygen consumption, once cells were reoxygenated. Expectedly, leukemic cell lines and primary patient blasts of a FLT3-WT genotype showed diminished capacity to consume oxygen after being kept at 1% oxygen for several days (Fig. 6B). In contrast, FLT3-ITD cell lines sustained their mitochondrial respiration capacity despite having adapted to low oxygen levels and were able to resume respiration immediately (Fig. 6C). To confirm the role of PDP1 in driving this OXPHOS switch, we performed the same experiment with prior PDP1-depletion in Molm13 cells (3 days before measurement). Here, we observed reduced OCR in siPDP1 treated cells upon re-oxygenation indicating at least partial responsibility of PDP1 for maintaining respiration capacity (Fig. 6D).

### PDP1 modulates responses to FLT3-inhibition and mediates resistance

**Fig. 7:**
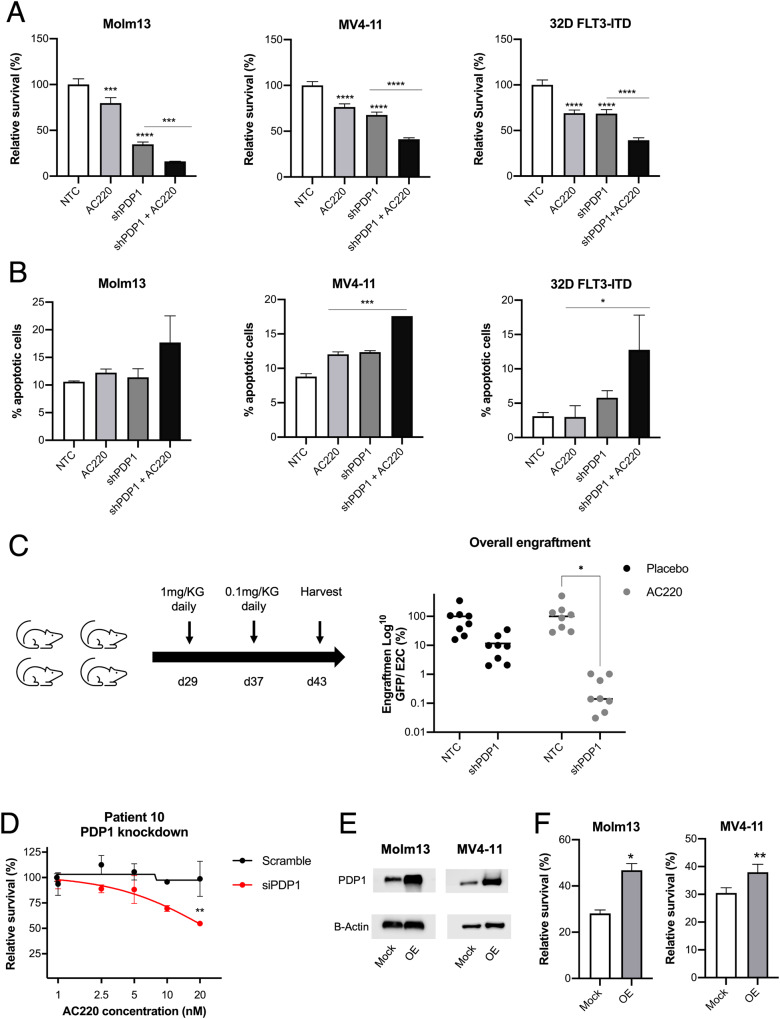
PDP1 is a resistance mechanism to FLT3-inhibition. **A** Combinatorial effect of PDP1 knockdown and FLT3-inhibition with 1 nM AC220 for 24 h in Molm13, MV4-11 and 32D FLT3-ITD cells (representative experiment, *n* = 2 biological replicates). **B** Apoptosis levels upon PDP1 knockdown in combination with AC220 treatment (1 nM for 24 h) (representative experiment, *n* = 2 biological replicates). **C** In vivo competition assay of GFP-positive NTC and shPDP1 MV4-11 cells mixed, each, in 1:1 ratio with E2C-positive NTC cells and transplanted into NSG mice (*n* = 8 per group) (5*10^5^ cell per mouse). Each group (NTC:NTC and NTC:shPDP1) was either treated with AC220 or DMSO (placebo) as shown in the scheme. Total engraftment is shown as a ratio between GFP and E2C. **D** AC220 sensitivity (48 h treatment) upon PDP1 knockdown using an siRNA approach in primary AML blasts from an AC220-resistant patient. This experiment was performed once on freshly aspirated peripheral blasts. **E** Western blot of PDP1 overexpression in Molm13 and MV4-11 cells. **F** AC220 sensitivity in Molm13 and MV4-11 cells upon PDP1 overexpression (1.5 nM AC220 for 72 h) (representative experiment, *n* = 2–3 biological replicates). Data are presented as mean values ± SD. In (**A**) and (**B**), one-way ANOVA with Bonferroni’s multiple comparisons was performed (****p ≤ 0.0001, ***p ≤ 0.001, *p ≤ 0.05). In (**C** and **D**), two-way ANOVA with Bonferroni’s multiple comparisons was performed (*p ≤ 0.05). In (**F**), unpaired Student’s *t*-test was performed (**p ≤ 0.01, *p ≤ 0.05).

In line with the well-established role of metabolism in drug sensitivity in AML and considering the central metabolic functions of PDP1, we aimed to investigate the relevance of PDP1 as a modulator of leukemia response to FLT3 inhibitors. First, we assessed the sensitivity of Molm13, MV4-11 and 32D FLT3-ITD cells to FLT3-inhibition with quizartinib (AC220) and compared the sensitivity with and without PDP1 knockdown. In all cases, PDP1 knockdown resulted in increased sensitivity towards AC220 (Fig. 7A). PDP1 knockdown primed FLT3-ITD cells to apoptosis when incubated with low doses of AC220 (Fig. 7B). To further validate these results, we performed an in vivo competition assay where we transplanted NSG mice with MV4-11 cells, transduced with either NTC or shPDP1 GFP-positive shRNA vector, mixed in a 1:1 ratio with MV4-11 cells transduced with an E2Crimson (E2C)-positive NTC shRNA vector. Both NTC and PDP1-KD cells engrafted, which was in accordance with the previous transplantation experiment (Fig. 4H). However, also in accordance with the previous transplantation experiment, PDP1 knockdown caused a significant competitive growth disadvantage in MV4-11 by day 43 when the leukemic mice were sacrificed. When we treated the engrafted mice with AC220 (from day 29), the PDP1-knockdown MV4-11 cells proved to be exquisitely sensitive to this treatment (Fig. 7C).

Furthermore, we investigated the potential of targeting PDP1 to sensitize AC220-resistant cells. We incubated bone marrow blasts from an FLT3-ITD-positive AML patient with clinical resistance to AC220 with siPDP1. As shown in Fig. 7D, clinical resistance correlated well with resistance to AC220 ex vivo. Strikingly, PDP1 knockdown significantly sensitized the cells and rendered them responsive to AC220 treatment (Fig. 7D). Conversely, to imitate the role of high PDP1 levels in resistance to AC220, we overexpressed PDP1 in the FLT3-ITD cell lines Molm13 and MV4-11 (Fig. 7E), which resulted in desensitization to AC220 mimicking a more resistant phenotype (Fig. 7F).

**Fig. 8:**
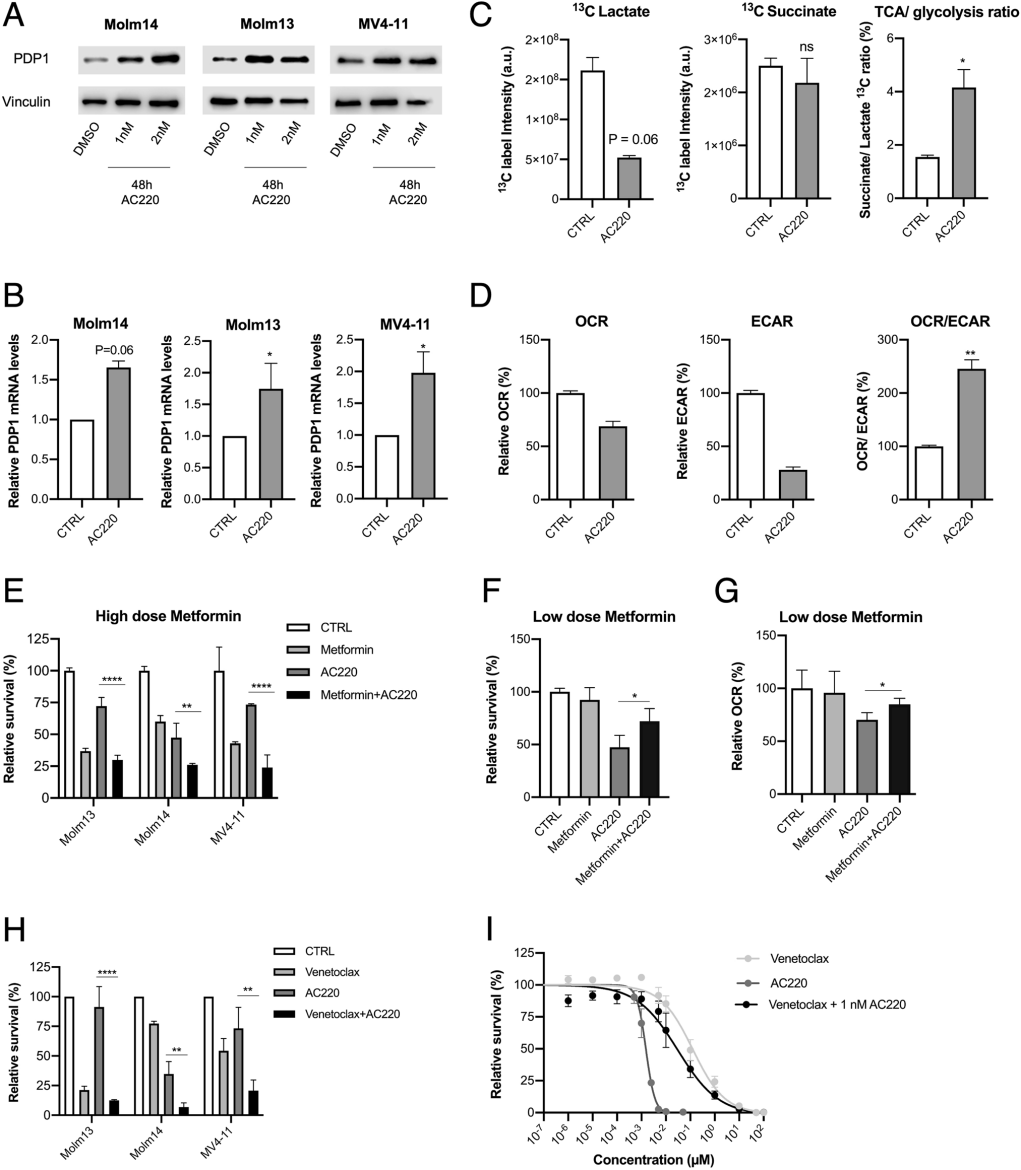
PDP1 is upregulated upon FLT3-inhibition as a survival strategy. **A** Western blot of PDP1 levels upon FLT3-inhibition. **B** qPCR measurements of PDP1 mRNA levels upon FLT3-inhibition (normalized to RPII levels) (*n* = 2–4). **C** NMR measurements of succinate and lactate ^13^C intensity and their ratio after 24 h of labelling with ^13^C glucose (*n* = 2 technical replicates). **D** Representative Seahorse measurements of OCR, ECAR and their ratio upon FLT3-inhibition (the experiment was repeated twice). **E** Synergism of high dose of metformin (5 mM) with AC220 (1 nM) (72 h treatment) (normalized to untreated control) (representative experiment is shown out of 3 biological replicates). **F** Low dose of metformin (50 μM) antagonizes the effects of AC220 (1 nM) in Molm14 cells (72 h treatment) (representative experiment is shown out of 2 biological replicates). **G** OCR measurements in Molm14 cells treated with AC220 (1 nM) and low dose metformin (50 μM) (24 h treatment) (representative experiment is shown out of 2 biological replicates). **H** Synergism of venetoclax (10 nM) and AC220 (1 nM) (72 h treatment) in FLT3-ITD AML cell lines (normalized to untreated control) (*n* = 3 biological replicates). **I** Dose response curve of venetoclax and AC220 in Molm14 cells. Venetoclax + AC220 combination was performed with a fixed concentration of AC220 (1 nM) (*n* = 3). Data are presented as mean values ± SD. In (**C**) and (**D**), unpaired Student’s *t*-test was performed (**p ≤ 0.01, *p ≤ 0.05). In (**E**–**H**), one-way ANOVA with Bonferroni’s multiple comparisons was performed (****p ≤ 0.0001, **p ≤ 0.01, *p ≤ 0.05).

Given this role of PDP1 in modulating AC220 responses, we examined the mechanism by which it exerts this function. Thus, we investigated PDP1 dynamics upon FLT3-inhibition for short periods (24–48 h). Unexpectedly, PDP1 was upregulated upon AC220 treatment in all of the FLT3-ITD AML cell lines on the protein level (Fig. 8A) as well as on the mRNA level (Fig. 8B). To investigate whether this upregulation has metabolic implications by further shifting the cells towards OXPHOS metabolism, we first performed a tracer-based NMR assay with ^13^C-glucose under AC220 treatment conditions. Expectedly, label incorporation analysis revealed higher succinate/lactate ^13^C-ratio upon FLT3-inhibition (Fig. 8C). Additionally, we assessed the OCR/ECAR ratio upon FLT3-inhibition as an indicator of the cellular metabolic status. Although treatment with AC220 reduced the overall metabolic activity, it affected ECAR more, resulting in a more prominent OXPHOS phenotype (Fig. 8D). Taken together, our data indicate that inhibition of FLT3-ITD signaling triggers strong upregulation of PDP1, inducing a pronounced OXPHOS metabolic profile that has a cytotoxicity-protective nature. If this was true, AC220-treated cells should be especially sensitive to OXPHOS inhibition. Therefore, we investigated a possible synergy between AC220 and mitochondrial targeting. Metformin is known to be a bidirectional effector of the mitochondrial respiration, which suppresses OXPHOS at high doses and conversely, enhances it at low concentrations [51]. In line with our hypothesis, an additive effect could be observed when AC220 was combined with high dose metformin (Fig. 8E). Conversely, an antagonizing effect was observed when AC220 was combined with low doses of metformin (50 μM) (Fig. 8F), which we showed to coincide with a metformin-driven increased OCR (Fig. 8G).

To further confirm this finding, we followed up by investigating responses to venetoclax, a BCL2 inhibitor effective in the treatment of AML patients unfit to receive intensive chemotherapy [54]. Since cellular sensitivity to venetoclax was shown to depend in part on the OXPHOS metabolic state of the cell [26, 27], we reasoned that AC220-induced OXPHOS would result in pharmacological synergism between venetoclax and AC220. Indeed, we demonstrated that AC220-induced metabolic changes render the cells vulnerable to venetoclax treatment and enhance its potency (Fig. 8H) and efficiency (Fig. 8I).

Altogether, these findings provide evidence that PDP1-driven OXPHOS metabolism may be a survival strategy upon FLT3-inhibition, and that this OXPHOS dependency can be exploited to increase the efficacy of FLT3 inhibition.

## Discussion

In this report, we provide evidence of an interdependence of FLT3-ITD in AML with PDP1, an enzyme responsible for the metabolic switch between aerobic glycolysis and oxidative glucose metabolism. We show that PDP1 is crucial for maintaining mitochondrial glucose oxidation in FLT3-ITD-positive AML, and that through this, PDP1 significantly contributes to its leukemic phenotype and its resilience against FLT3 inhibition.

We present a thorough analysis of the fate of glucose in FLT3-ITD versus FLT3-WT AML cell line models. NMR studies allowed us to directly decipher the fate of exogenous glucose and so to directly demonstrate that FLT3-ITD alters the way how glucose is metabolized. The results are in line with the more general notion that lactate-producing glycolysis does not substitute for mitochondrial pyruvate metabolism, but rather supplements it in cancer cells [15, 16]. Our data are also in line with several reports that indicate that maintaining mitochondrial metabolism is not just an observable phenomenon, but an important metabolic feature of AML, representing a possible vulnerability [28–30, 55]. Previously, it was reported that PDP1 expression and function are repressed in certain types of cancers, including AML [34, 35]. Our results paint a more heterogeneous picture, indicating that PDP1 expression may be differentially regulated by different AML driver lesions. Along these lines, it was recently reported that the sensitivity of AML cases to inhibition of complex I of the ETC is very heterogeneous, and among the more sensitive cases were those harboring an FLT3 mutation [24]. Moreover, another recent report has shown that, while most of FLT3-WT AMLs express high levels of PDK1 (the antagonizing kinase to PDP1 that drives glycolytic metabolism), FLT3-ITD AMLs are amongst the lowest PDK1-expresors, which coincides with their high OXPHOS state [56]. In our study, we establish that increased OXPHOS activity is a metabolic feature of FLT3-ITD-positive AML, and we link increased OXPHOS activity to an FLT3-ITD/PDP1 signaling axis. The endogenous tagging of PDP1 was a valuable tool for providing data on the role of signaling nodes downstream of FLT3-ITD that mediate the effect of FLT3-ITD on pyruvate metabolism. Previous work by us and others [3, 57] highlighted the importance of the RAS pathway in the oncogenic signaling and transformation of FLT3-ITD-positive leukemia and in this study, we extend this to its role in maintaining PDP1-driven OXPHOS. Along these lines, a recent study has demonstrated the efficacy of inhibiting mitochondrial metabolism against RAS-mutated AML [58]. These findings highlight the importance of future research on the crosstalk of FLT3-ITD & RAS pathways as well as the potential cooperativity of RAS inhibition and TKIs treatment.

We found it particularly interesting that FLT3-ITD-positive cells sustained their dependency on PDP1 even under prolonged hypoxic conditions. It is widely accepted that in order to survive in vivo, AML cells, and especially leukemia initiating cells or stem cells (LSCs) that give rise to disease relapse, have to rapidly adapt to changing metabolic conditions [59–63], one of which is that they have to be able to travel between niches with normoxic and highly hypoxic conditions [64–66]. The ability to jump-start OXPHOS as soon as the cells are provided with oxygen provides an additional metabolic flexibility that may enable FLT3-ITD-positive AML cells to rapidly resume their aggressive growth properties when faced with such demanding variations of oxygen availability in AML bone marrow.

FLT3-ITD maintains OXPHOS through a PDP1-mediated boost of the TCA cycle. Besides its function in pyruvate metabolism, PDP1 has been shown to directly or indirectly influence a number of other essential mitochondrial regulatory and metabolic switches such as energy production and mTOR activation [67, 68], lipid biosynthesis [36] and redox sensing [69]. Our data indicate that whatever the functions of PDP1 may be, its role to sustain AML cell survival is dependent on its ability to boost OXPHOS activity. Proliferation and survival of Rho Zero cells that are adapted to survive in the absence of a functional ETC do not depend on PDP1 for their proliferation and survival. Moreover, whatever means we used to pharmacologically inhibit the ETC in FLT3-ITD cells, it synergized with the inhibition of FLT3 to interrupt cellular survival and proliferation.

Our data demonstrate a role of PDP1 in the response of FLT3-ITD-dependent AML cells to FLT3-inhibition. FLT3-ITD-associated PDP1 overexpression stimulates OXPHOS, a metabolic pathway that mediates AML resistance against cytotoxic events like chemotherapy such as Ara-C, or tyrosine kinase inhibitor (TKI) treatment [25, 70–72]. Our data confirm that upon the removal of FLT3 activity, the oxygen consumption rate is maintained, showing for the first time that this is dependent on sustained PDP1 expression. In the initial experiments (Fig. 2), we showed that PDP1 expression is enhanced in FLT3-ITD-dependent leukemia cells, and thus it seemed at first sight surprising that FLT3 inhibition enhanced PDP1 expression in these cells even further. We assume that enhanced or sustained PDP1 expression is a general response to growth factor or oncogene signal deprivation. Indeed, it has been shown previously that mTOR inhibition, which is a well-known consequence of FLT3-ITD inhibition [73] in FLT3-dependent cells, causes a metabolic shift towards OXPHOS [74]. While our paper does not address how acute deprivation from oncogenic FLT3-ITD signals causes enhanced or sustained PDP1 expression, we do present data that PDP1 expression is involved in mounting resistance against AC220. In combination with recent results from another study, where FLT3-ITD positive cells escaped TKI treatment by increasing their glutamine channeling into the TCA cycle [41], one might speculate that indeed enhanced TCA cycle turnover and OXPHOS may be an interesting, potentially targetable mechanism of resistance. In line with this, we also observed in our settings that FLT3-ITD positive cells try to compensate for PDP1 depletion by rewiring their glutamine metabolism towards the TCA cycle (Supplementary Fig. 4). Our experiment, where we could restore sensitivity towards AC220 by PDP1 knockdown in primary patient AML blasts ex vivo, also points to the possibility that we may describe a real-world clinically relevant mechanism of secondary resistance towards FLT3 inhibitors, a phenomenon which occurs almost uniformly after weeks to months of monotherapy with these drugs [9, 10]. Further research into targeting the mitochondrial metabolism to overcome drug resistance, especially in FLT3-ITD-positive cases, therefore seems very promising.

In summary, we decipher PDP1 as a key metabolic gatekeeper in FLT3-ITD-positive AML cells and demonstrate its essential role in their proliferation, survival and drug responses. The novel findings of this study open a new horizon for the therapeutic targeting of PDP1 or its downstream functions in FLT3-ITD-driven AML.

### Supplementary information

Supplementary Information

## Data Availability

For detailed information regarding patients’ information, reagents, western blotting, NMR tracer-based assays and CRISPR screen validation, see Supplementary Information. CRISPR screen results can be found in the Supplementary Files.
