# Supplementary material for: PDP1 is a key metabolic gatekeeper and modulator of drug resistance in FLT3-ITD-positive acute myeloid leukemia
Source: Leukemia. 2023 Nov 7;37(12):2367–82. doi: 10.1038/s41375-023-02041-5 (PMC10681906; doi:10.1038/s41375-023-02041-5)
Supplement: Supplementary file 1 — Supplementary Information [file 41375_2023_2041_MOESM1_ESM.docx]

**PDP1 is a key metabolic gatekeeper and modulator of drug resistance in FLT3-ITD-positive acute myeloid leukemia**

**Authors:** Islam Alshamleh^1,2,3,4^, Nina Kurrle^2,3,4^, Philipp Makowka^3^, Raj Bhayadia^5^, Rahul Kumar^6^, Sebastian Süsser^3^, Marcel Seibert^2,3,4^, Damian Ludig^1^, Sebastian Wolf^3,4^, Sebastian E. Koschade^3^, Karoline Stoschek^3^, Johanna Kreitz^3,4^, Dominik Fuhrmann^7^, Rosa Toenges^3^, Marco Notaro ^8^, Federico Comoglio^8^, Jan Jacob Schuringa^9^, Tobias Berg^3,10^, Bernhard Brüne^4,7,11^, Daniela S. Krause^4,6,12^, Jan-Henning Klusmann^5^, Thomas Oellerich^2,3,4^, Frank Schnütgen*^2,3,4^, Harald Schwalbe*^1,2,4^ and Hubert Serve*^2,3,4^

^1^ Center for Biomolecular Magnetic Resonance (BMRZ), Institute of Organic Chemistry and Chemical Biology, Johann Wolfgang Goethe University, 60438 Frankfurt am Main, Germany.

^2^ German Cancer Consortium (DKTK), partner site Frankfurt/Mainz, and German Cancer Research Center (DKFZ), Heidelberg, Germany.
^3^ Department of Medicine, Hematology/Oncology, Johann Wolfgang Goethe University, 60590 Frankfurt, Germany.
^4^ Frankfurt Cancer Institute, Johann Wolfgang Goethe University, 60596 Frankfurt, Germany.

^5^ Department of Pediatrics I, Johann Wolfgang Goethe University, 60590 Frankfurt, Germany.

^6^ Georg-Speyer-Haus, Institute for Tumor Biology and Experimental Therapy, 60596, Frankfurt am Main, Germany.

^7^ Institute of Biochemistry I, Faculty of Medicine, Johann Wolfgang Goethe University, 60590 Frankfurt am Main, Germany.

^8^ enGene Statistics GmbH, Basel, Switzerland.

^9^ Department of Experimental Hematology, University Medical Center Groningen, University of Groningen, Groningen, The Netherlands.

^10^ Centre for Discovery in Cancer Research and Department of Oncology, McMaster University, Hamilton, Ontario, Canada.

^11^ Project Group Translational Medicine and Pharmacology TMP, Fraunhofer Institute for Molecular Biology and Applied Ecology, 60596 Frankfurt am Main, Germany.

^12^ Georg-Speyer-Haus; German Cancer Consortium (DKTK), partner site Frankfurt/Mainz, and German Cancer Research Center (DKFZ), Heidelberg, Germany.

* Corresponding authors.

**Corresponding authors:**

*Corresponding author: Tel: +49 69 6301 4941, E-mail: schnuetgen@em.uni-frankfurt.de

*Corresponding author: Tel: +49 69 7982 9737, E-mail: schwalbe@nmr.uni-frankfurt.de

*Corresponding author: Tel: +49 69 6301 4634, E-mail: serve@em.uni-frankfurt.de

**Supplemental Figures**


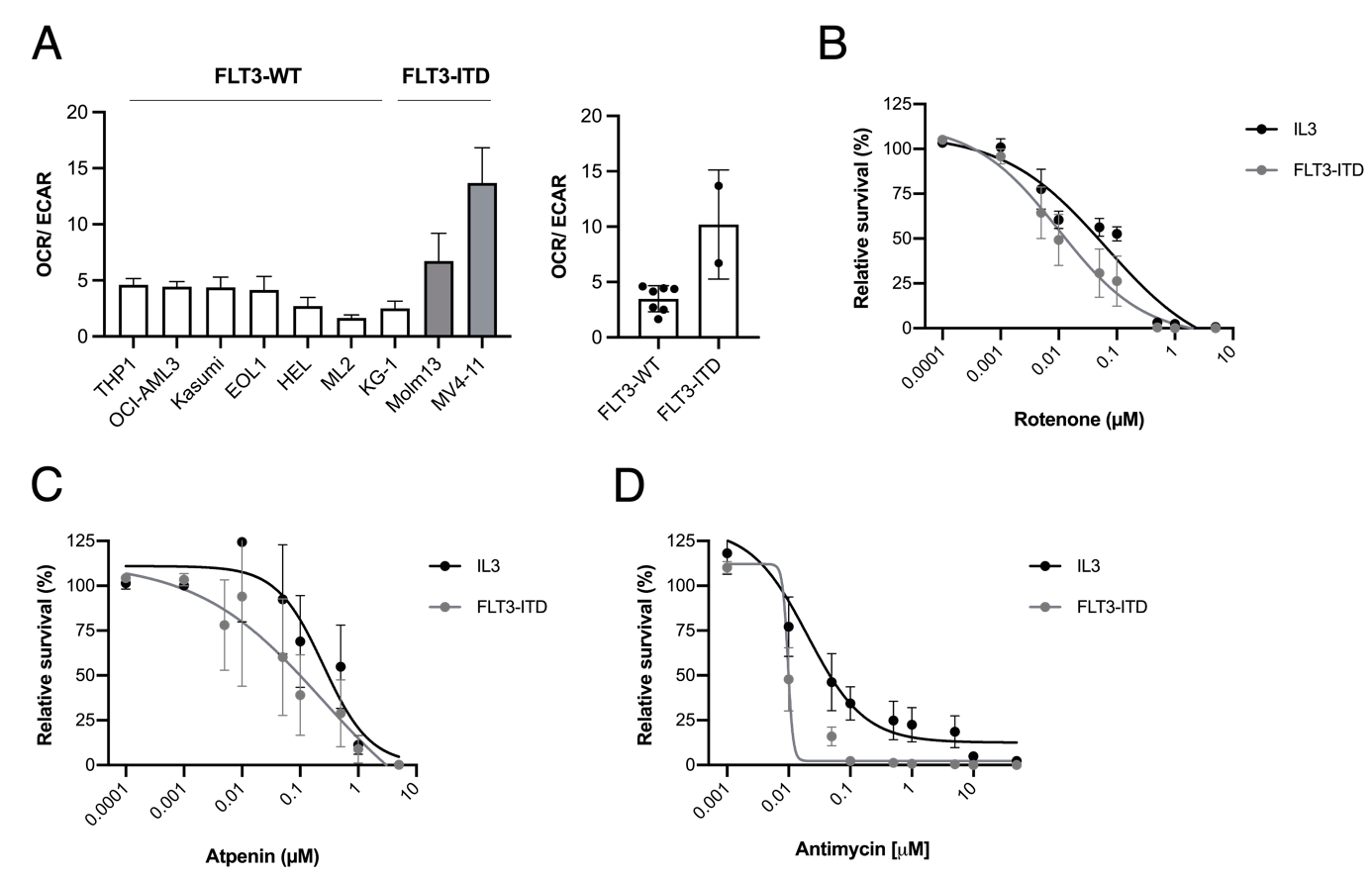


**Supplementary Figure 1: FLT3-ITD-positive cells respire more, and they are more sensitive to mitochondrial inhibition.** A. Seahorse measurements of OCR/ ECAR ratio in a panel of FLT3-ITD-positive AML cell lines (n=2-4). IC_50_ curves of different B. Rotenone, C. Atpenin A and D. Antimycin A5 sensitivities in 32D cells under FLT3-ITD signaling in comparison to IL3 (n=3).


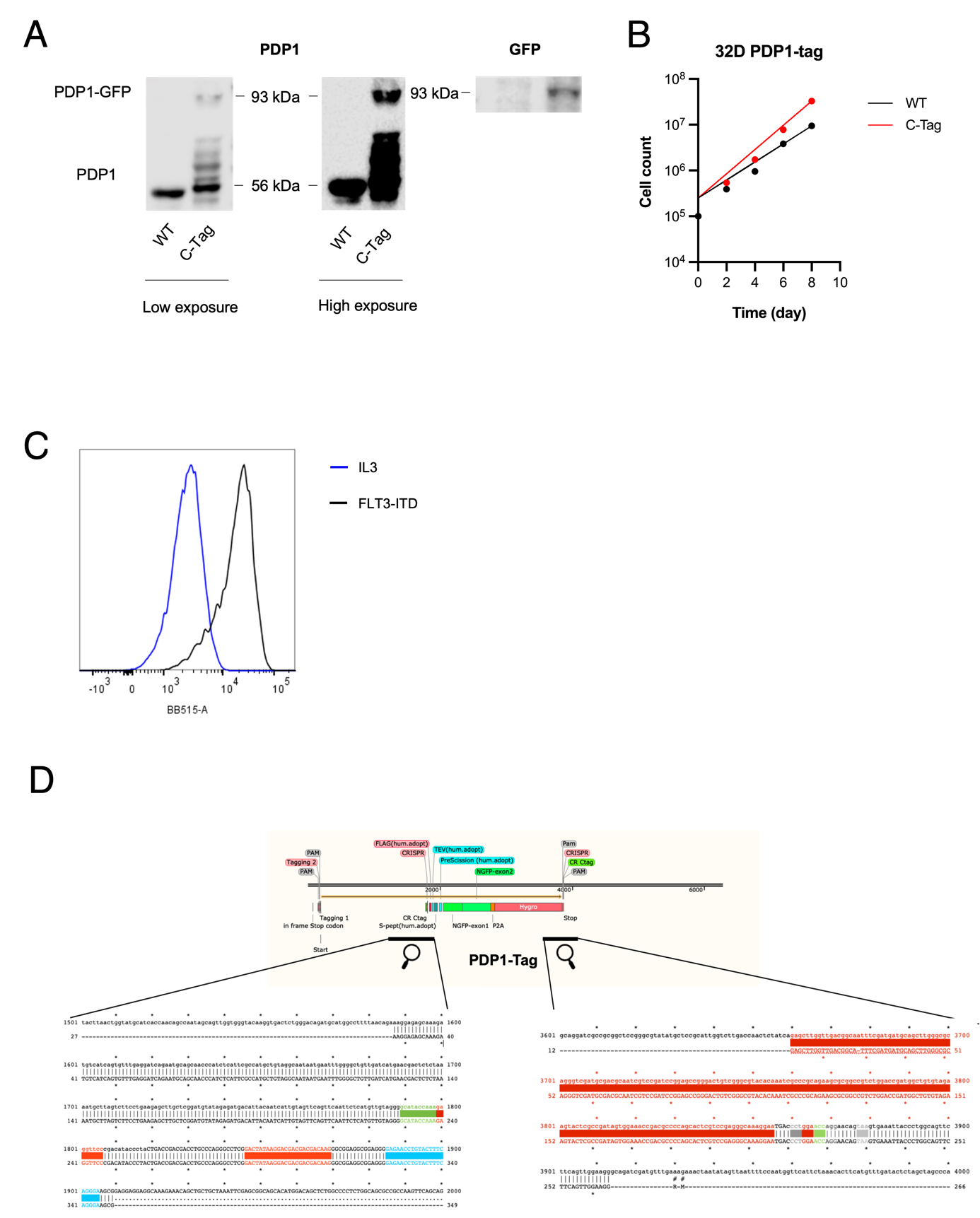


**Supplementary Figure 2: Endogenous tagging of PDP1.** A. Western blot of PDP1 (WT and fusion protein) detected with PDP1 antibody (two left blots) and GFP antibody (right blot). Bands observed on the tagged PDP1 blot (between 56-83 kDa) are likely degradation products of the fusion protein. B. Cumulative growth assays of PDP1-tagged cells compared to their WT counterparts. C. Flowcytometry analysis of GFP intensity in PDP1-tagged 32D cells under grown with either IL3 or FLT3-ITD signaling. D. Sequencing results of the PDP1-tag region in 32D cells (aligned against the designed vector sequence (top row)).


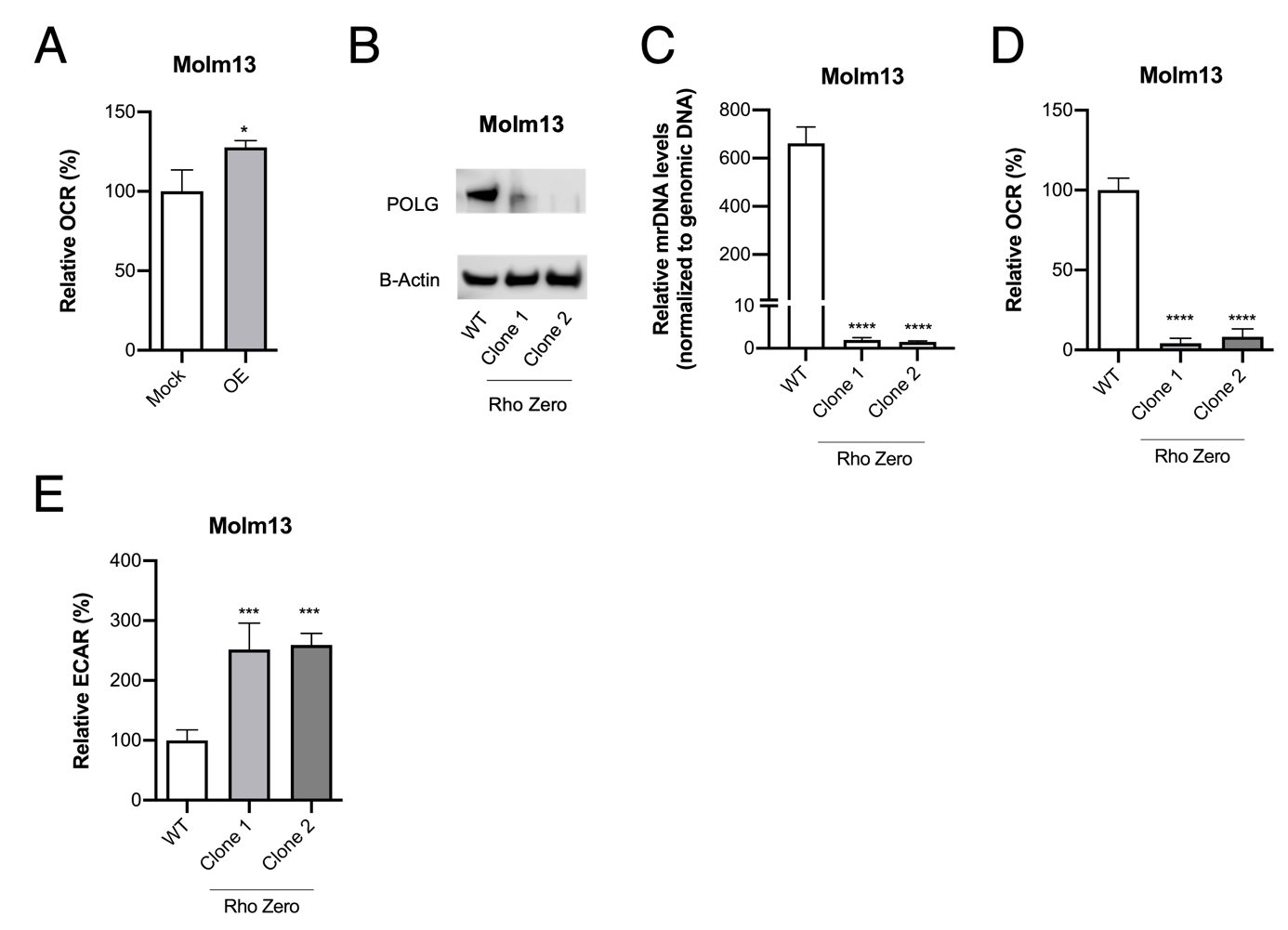


**Supplementary Figure 3: Mitochondrial respiration in Molm13 cells with PDP1 overexpression or in Rho Zero Molm13 cells lacking mitochondrial DNA.** A. Seahorse measurements of respiration capacity (OCR) in Molm13 cells with PDP1 overexpression (n=2, and the experiment is plotted for 4 technical replicates). B. Western blot of mitochondrial polymerase γ (POLG) knockout in Molm13 Rho Zero cells. C. Mitochondrial DNA (mtDNA) levels in Molm13 Rho Zero cells (assessed by ND1 levels using real-time PCR) (n=2) D. Seahorse measurements of respiration capacity (OCR) and E. glycolytic activity (ECAR) in Molm13 Rho Zero cells (n=4 technical replicates).

**
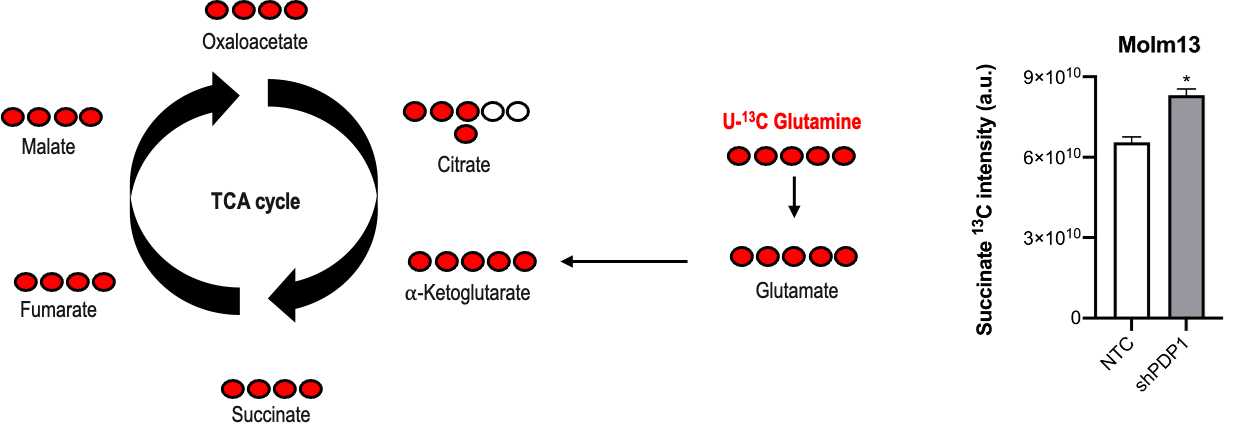
**

**Supplementary Figure 4: FLT3-ITD-positive cells adapt to PDP1 knockdown by enhancing glutamine shuttling into the TCA cycle.** Succinate ^13^C-label intensity in Molm13 cells grown in media containing ^13^C uniformly labelled glutamine upon PDP1 knockdown. Data are presented as mean values ± SD. Unpaired Student’s t-test was performed, * p ≤ 0.05 (n=2 technical replicates).

**Supplemental methods**

Inhibitors, reagents and chemicals

AC220 and venetoclax were bought from Selleckchem. HEPES buffer, rotenone, metformin, uridine, polyethylenimine (PEI) and ^13^C-labelled glucose were bought from Sigma-Aldrich. Puromycin was bought from InvivoGen. ^13^C-labelled glutamine was bought from Eurisotop. Atpenine A5 and Antimycin A were bought from Abcam. Sodium dichloroacetate was bought from Santa Cruz. BAY-294 negative control (#6906) and BAY-293 (#6857) were bought from Tocris.

Synergy assays

For detection of synergism, 25,000 cells/well for AML cells and 10,000 cells/well for 32D cells were seeded with increasing concentrations of AC220 and venetoclax arranged in a checkerboard pattern. CellTiter-Glo measurements were performed 72 hours after treatment. Dose-response curves, IC_50_ values and analysis of fixed combinations could be analyzed from these data. Dose-response curve fitting was performed with robust regression.

Preparation of protein lysates, SDS-Page and Western blotting

Protein lysates were harvested with RIPA buffer (150mM sodium chloride, 1% Triton X-100, 0.5% sodium deoxycholate, 0.1% SDS (sodium dodecyl sulfate) and 50 mM Tris-HCl, PH=7.5). RIPA buffer was freshly supplemented with EDTA-free protease inhibitor (Merck), 5 mM NaF (Sigma-Aldrich) and Pierce Nuclease (ThermoFisher). 1*10^6^ cells were lysed with the buffer, vortexed and incubated on ice for 20 minutes followed by 5 min incubation at 37°C and then centrifuged for 10 minutes at 15000 x g/4°C. Protein concentration was measured using Bradford assay (5X Roti-Quant reagent, Carl Roth) and lysates were prepared with 4X loading dye (Carl Roth) and boiled for 5 minutes at 94°C. Protein lysates were run on a 10% agarose gel using MOPS SDS running buffer, then blotted on a nitrocellulose membrane (0.2 μM pore size, Bio-Rad). Primary antibodies (Supplemental Table 1) were applied overnight at 4°C. Secondary antibodies (Supplemental Table 1) were applied for 1 hour at room temperature and then signals were visualized on a LI-COR (Biosciences GmbH) using SuperSignal™ West Femto chemiluminescence substrate (ThermoFisher). Image quantification was done using Image Studio.

Supplementary Table 1: Antibodies information.

| Antibody | Biological source | Provider | Identifier or catalogue number |
| --- | --- | --- | --- |
| PDP1 (primary) | Rabbit | Cell Signaling | 65575 |
| POLG (primary) | Rabbit | Cell Signaling | 13609 |
| Vinculin (primary) | Mouse | Sigma-Aldrich | V9131 |
| B-actin (primary) | Mouse | Sigma-Aldrich | 4970 |
| PDHA1 (primary) | Mouse | Molecular Probes | A21323 |
| pPDHA1 S293 (primary) | Rabbit | Merck | ABS204 |
| eGFP (primary) | Mouse | Roche | 11814460001 |
| HRP anti-mouse (secondary) | Goat | Santa Cruz | sc-2005 |
| HRP anti-rabbit (secondary) | Goat | Santa Cruz | sc-2004 |

Rho Zero cells clone validation

Subclones were generated by single cell sorting, and the knockout was confirmed on genomic and protein levels before using them for further analysis. The loss of mtDNA was confirmed by quantitative real-time PCR on genomic DNA using the mitochondrial genes ND1 and ND2 as target genes on mtDNA and 18S rRNA, ACTB and COX6A1 as endogenous controls on nuclear DNA (1). The resulting cells could be maintained in media supplemented with uridine and pyruvate as reported previously for the human osteosarcoma cell line 143B.TK- (2).

Tracer-based assay

For labelling experiments, cells were plated 24 hours before extraction in glucose-free or glutamine-free media and supplemented with 11 mM uniformly ^13^C-labelled glucose or 2 mM uniformly ^13^C-labelled glutamine. Samples were processed similarly to the 1D extracts. ^1^H–^13^C HSQC spectra were recorded for glucose-labelled samples on a Bruker 600MHz AVIIIHD spectrometer (5mm TCI Prodigy probe head) using the standard Bruker pulse program hsqcetgpsp that implements an echo/anti-echo time-proportional phase incrementation gradient selection. 1024 points were recorded in the direct dimension, 2048 points in the indirect dimension with 10% NUS function and a spectral width of 169 ppm, measured at 25°C. Glutamine labelled samples were recorded on a a Bruker 600MHz AV neo spectrometer (1.7mm TCI cryoprobe head) using the pulse program hsqcctphprsp (constant-time HSQC). 1428 points were recorded in the direct dimension, 1468 points in the indirect dimension with 10% NUS function and a spectral width of 95 ppm, measured at 25°C. Metabolites identification and quantification was performed using Chenomx, Metabolab (3) and Sparky (UCSF) (4).

Validation of CRISPR screen results

PDP1-tagged 32D cells were adapted to FLT3-ITD signaling (by mIL3 withdrawal) and reselected using 5 µg/mL blasticidin (Invivogen) and 200 µg/mL hygromycin (Corning). 1.5*105 cells were transduced with sgRNAs targeting GRB2 or SOS1 and then analyzed by flowcytometry on day 3, 4 and 5 post transduction. Mean fluorescence intensity (MFI) of GFP signal (PDP1 C-terminal) was measured in the E2-Crimson-positive cells (cells that had GRB2 or SOS1 knockout) on day 3, 4 and 5 post transduction.

Supplementary Table 2: Patient details of primary AML blasts samples.

| Number | Sex | Age at diagnosis | % BM blasts at diagnosis | WBC/nl at diagnosis | FAB subtype | Karyotype | Molecular genetics |
| --- | --- | --- | --- | --- | --- | --- | --- |
| 1 | w | 50 | 38 | 18,51 | M2 | n.a. | ASXL1, DNMT3A, IDH2 |
| 2 | m | 53 | 66 | 53 | M1 | 46,XY | NPM1, FLT3-ITD |
| 3 | m | 83 | 34 | 27,22 | M4 | 46,XY | CEBPA |
| 4 | w | 41 | 52 | 3,01 | M4eo | 46 XX, Inv(16)(p13q22), 47 XX,Inv(16)(p13q22)+22 | CBFB-MYH11 |
| 5 | m | 55 | 58,00 | 12,55 | M2 | 46,XY | NPM1 |
| 6 | w | 73 | 81 | 165,29 | M4 | 46,XX | NPM1, FLT3-ITD |
| 7 | m | 73 | 80 | 104,49 | M1/2 | 46,XY | NPM1, FLT3-ITD |
| 8 | m | 77 | 90 | 130,00 | M4 | 46,XY | NPM1, FLT3-ITD, CEBPA |
| 9 | w | 46 | 90 | 105,30 | n.a. | 46,XX | NPM1, FLT3-ITD |
| 10 | m | 46 | 60 | 207,74 | M4 | 46, XY | NPM1, FLT3-ITD |
| 11 | m | 40 | 73,00 | 3,02 | n.a. | 46, XY ish del(17)q11q11 [20] | NPM1, FLT3-ITD, IDH1, IDH2 |
| 12 | m | 65 | 83,00 | 158 | n.a. | 46, XY [20] | NPM1, FLT3-ITD, FLT3-TKD |
| 13 | w | 46 | 80,00 | 63 | M2 | n.a. | NPM1, FLT3-ITD, CEBPA |
| 14 | w | 68 | 90,00 | 143 | M2 | 46,XX [20] | NPM1, FLT3-ITD |
| 15 | m | 59 | 90,00 | 491 | M1 | 46, XY [5] | NPM1, FLT3-ITD |
| 16 | m | 46 | 60 | 207,74 | M4 | 46, XY | NPM1,  FLT3-ITD |
| 17 | w | 59 | 40,00 | 93 | n.a. | 46,XX [20] | NPM1, FLT3-ITD, DNMT3A, NRAS |
| 18 | w | 48 | 31,00 | 21 | n.a. | 46, XX | NPM1, FLT3-ITD, IDH1, IDH2 |
| 19 | w | 57 | 87,00 | 8,46 | n.a. | 46, XX | FLT3-ITD, KMT2A-PTD |
| 20 | m | 39 | 49,00 | 36 | M5 | 46,XY [20] | NPM1 |
| 21 | m | 41 | 65,00 | 11,9 | AML NOS | 46, XY [20] | - |
| 22 | m | 73 | 50,00 | 50 | AML after MDS | 45,XY,-7 [9]; 45,XY,del(2)(q32q35),-7 [11] | - |
| 23 | m | 29 | 80,00 | 3,12 | AML NOS | 47,XY, +4, del(5)(q12q15),t(7;14)(q21;q32) [14], 46,XY[6] | - |
| 24 | m | 72 | 46,00 | 90,9 | sAML after CMML | 49,XY,+4,+7,+8 [5]; 46,XY [19] | - |
| 25 | m | 77 | 30,00 | 239 | M5 | 46,XY | - |
| 26 | w | 23 | 66,00 | 39 | n.a. | t(5,11)(q35;p15) | FLT3-ITD, NUP98-NSD1-rearrangement |
| 27 | m | 51 | 70,00 | 16,09 | n.a. | 46, XY [20] | CEBPA |
| 28 | w | 70 | 21,00 | 1,2 | AML with myelodysplasia related changes | 46XX | KMT2A (MLL)- PTD, IDH2 |

Patient 10 received two cycles of intensive induction chemotherapy in combination with Midostaurin, without achieving remission. As bridge to transplant therapy, he received quizartinib for 3 months, initially 30 mg/day followed by dose escalation (60 mg/day) after one month. Within that time, he achieved blast clearance after two months in the peripheral blood, however in the bone marrow his blast count only decreased from 42% to 16%.

Supplementary Table 3: PDP1 shRNA sequences.

| shRNA | Sequence (5’ – 3’) |
| --- | --- |
| Human shPDP1 (1) (TRCN0000356073) | CCGGCACTGGTGAGTCGACTGATATCTCGAGATATCAGTCGACTCACCAGTGTTTTTG |
| Human shPDP1 (2) (TRCN0000003081) | CCGGGCTGTGTGTAGTCTCTTGGTTCTCGAGAACCAAGAGACTACACACAGCTTTTT |
| Mouse shPDP1 (1) (TRCN0000081381) | CCGGGCAGAATCTATGGCACTGCATCTCGAGATGCAGTGCCATAGATTCTGCTTTTTG |
| Mouse shPDP1 (2) (TRCN0000081382) | CCGGCGCCATGCTGTAGGCAATAATCTCGAGATTATTGCCTACAGCATGGCGTTTTTG |

Cloning of gRNAs into CRISPR vector and endogenous tagging

sgRNAs that target the C-Terminus of PDP1 were cloned into a plenti-CRISPR-vE2-Crimson-Cas9 vector (Addgene) using Golden Gate reaction (5 µl of each sense and antisense oligos (100 µM stocks, Sigma-Aldrich) (sequences in supplementary Table 4) were mixed and heated up to 95 °C for 5 min and cooled down to RT again to anneal, then added to a mixture of 250 ng of the vector, 2 µl of ligase buffer, 1 µl of BsmBI0 restriction enzyme, 1 µl of ligase and 15 µl of water followed by a Golden Gate protocol (37°C for 5 min, 16°C for 10 min (10 cycles), 37°C for 15 min and 80°C for 5 min). The tagging vector used is: pTarg_Clap_-1_-DTA+gRNA (C-Term.). Both vectors were heat shock transformed into DH5 alpha competent E. coli (30 min on ice, followed by 45 seconds at 42°C then back on ice for 2 min), and the plasmids were extracted using a mini-prep reaction (QAIprep®, Qaigen) and sequence by Microsynth Seqlab.

Supplementary Table 4: Sequences of sgRNAs oligonucleotides.

| Oligonucleotides | Sequences (5’-3’) |
| --- | --- |
| Mouse PDP1 C-tag (sense) | CACCGGTAGGGGCATACCAAAACC |
| Mouse PDP1 C-tag (antisense) | AAACGGTTTTGGTATGCCCCTACC |
| Human & Mouse SOS1 (sg1) (sense) | CACCGAACTTAGTGTAAAGTTACT |
| Human & Mouse SOS1 (sg1) (antisense) | AAACAGTAACTTTACACTAAGTTC |
| Human & Mouse SOS1 (sg2) (sense) | CACCGTATGTAAACAGAAACCTGG |
| Human & Mouse SOS1 (sg2) (antisense) | AAACCCAGGTTTCTGTTTACATAC |
| Human & Mouse GRB2 (sg1) (sense) | CACCGTTGAAGTCATATTTGGCGA |
| Human & Mouse GRB2 (sg1) (antisense) | AAACTCGCCAAATATGACTTCAAC |
| Human & Mouse GRB2 (sg2) (sense) | CACCGGAGCTGAGCTTCAAAAGGG |
| Human & Mouse GRB2 (sg2) (antisense) | AAACCCCTTTTGAAGCTCAGCTCC |

To confirm correct tag integration on the genetic level in the 32D cells, genomic DNA was extracted (Phenol/Chloroform/Isoamyl-alcohol-based protocol) and PCR reactions were performed using primers that cover both sides of the PDP1-GFP tagging region as well as with primers for the WT allele. The PCR reaction contained 300 ng DNA, 5 µl 10X DreamTaq Buffer, 2 µl NTP, 2,5 µl of each primer (10 µM stock) (sequences in supplementary Table 5), 0,5 µl DreamTaq Polymerase and 35 µl ddH_2_O) and was run after the following protocol: 98°C for 1min, 32 cycles of (98°C for 30 seconds, 57°C for 30 seconds, 72°C for 30 seconds), 72C° for 5 min, then quenched at 12°C. Samples were sequenced by Microsynth Seqlab.

Supplementary Table 5: Primers sequences used for validating correct PDP1 tag integration.

| Primer | Sequence |
| --- | --- |
| PDP1 WT control | GATCTGCCCTTCCAACTGAA |
| Mouse PDP1 C-tag (sense) | AGCAGTTGGTGGGTACAAGG |
| Mouse PDP1 C-tag (antisense) | CACTTCCAGGCTGCTGAACT |
| Mouse PDP1 C-tag (sense) (2nd) | TCCTGACTTCATGAATGGTTG |
| Mouse PDP1 C-tag (antisense) (2nd) | ATATGCAGGATGGGGTGTGT |

DNA extraction for the CRISPR screen, sgRNA amplification and Next Generation Sequencing and clean up

DNA was extracted (same as above) and PCR reactions were then performed to attach P5 and P7 index primers to the sgRNAs sequences and amplify them (Sequences in supplementary Table 6). AMPure beads were used to clean up the DNA samples which were then loaded on an agarose gel and extracted from the bands to maximize DNA purity. Finally, PCR reactions of sgRNAs were then performed for Illumina sequencing.

Supplementary Table 6: Primers sequences used for amplification and indexing of sgRNAs.

| Primers | Sequences 5’-3’ |
| --- | --- |
| ILMN8_i5_1 | AATGATACGGCGACCACCGAGATCTAGCGCTAGACACTCTTTCCCTACACGACGCTCTTCCGATCTTTGTGGAAAGGACGAAACACCG |
| ILMN8_i5_2 | AATGATACGGCGACCACCGAGATCTGATATCGAACACTCTTTCCCTACACGACGCTCTTCCGATCTCTTGTGGAAAGGACGAAACACCG |
| ILMN8_i5_3 | AATGATACGGCGACCACCGAGATCTCGCAGACGACACTCTTTCCCTACACGACGCTCTTCCGATCTGCTTGTGGAAAGGACGAAACACCG |
| ILMN8_i5_4 | AATGATACGGCGACCACCGAGATCTTATGAGTAACACTCTTTCCCTACACGACGCTCTTCCGATCTAGCTTGTGGAAAGGACGAAACACCG |
| ILMN8_i5_5 | AATGATACGGCGACCACCGAGATCTAGGTGCGTACACTCTTTCCCTACACGACGCTCTTCCGATCTCAACTTGTGGAAAGGACGAAACACCG |
| ILMN8_i5_6 | AATGATACGGCGACCACCGAGATCTGAACATACACACTCTTTCCCTACACGACGCTCTTCCGATCTGCACCTTGTGGAAAGGACGAAACACCG |
| ILMN8_i5_7 | AATGATACGGCGACCACCGAGATCTACATAGCGACACTCTTTCCCTACACGACGCTCTTCCGATCTTGCACCTTGTGGAAAGGACGAAACACCG |
| ILMN8_i5_8 | AATGATACGGCGACCACCGAGATCTGTGCGATAACACTCTTTCCCTACACGACGCTCTTCCGATCTACGCAACTTGTGGAAAGGACGAAACACCG |
| ILMN8_i5_9 | AATGATACGGCGACCACCGAGATCTCCAACAGAACACTCTTTCCCTACACGACGCTCTTCCGATCTGAAGACCCTTGTGGAAAGGACGAAACACCG |
| ILMN8_i7_1 | CAAGCAGAAGACGGCATACGAGATCCGCGGTTGTGACTGGAGTTCAGACGTGTGCTCTTCCGATCTTCTACTATTCTTTCCCCTGCACTGT |
| ILMN8_i7_2 | CAAGCAGAAGACGGCATACGAGATTTATAACCGTGACTGGAGTTCAGACGTGTGCTCTTCCGATCTTCTACTATTCTTTCCCCTGCACTGT |
| ILMN8_i7_3 | CAAGCAGAAGACGGCATACGAGATGGACTTGGGTGACTGGAGTTCAGACGTGTGCTCTTCCGATCTTCTACTATTCTTTCCCCTGCACTGT |
| ILMN8_i7_4 | CAAGCAGAAGACGGCATACGAGATAAGTCCAAGTGACTGGAGTTCAGACGTGTGCTCTTCCGATCTTCTACTATTCTTTCCCCTGCACTGT |
| ILMN8_i7_5 | CAAGCAGAAGACGGCATACGAGATATCCACTGGTGACTGGAGTTCAGACGTGTGCTCTTCCGATCTTCTACTATTCTTTCCCCTGCACTGT |
| ILMN8_i7_6 | CAAGCAGAAGACGGCATACGAGATGCTTGTCAGTGACTGGAGTTCAGACGTGTGCTCTTCCGATCTTCTACTATTCTTTCCCCTGCACTGT |
| ILMN8_i7_7 | CAAGCAGAAGACGGCATACGAGATCAAGCTAGGTGACTGGAGTTCAGACGTGTGCTCTTCCGATCTTCTACTATTCTTTCCCCTGCACTGT |
| ILMN8_i7_8 | CAAGCAGAAGACGGCATACGAGATTGGATCGAGTGACTGGAGTTCAGACGTGTGCTCTTCCGATCTTCTACTATTCTTTCCCCTGCACTGT |
| ILMN8_i7_9 | CAAGCAGAAGACGGCATACGAGATAGTTCAGGGTGACTGGAGTTCAGACGTGTGCTCTTCCGATCTTCTACTATTCTTTCCCCTGCACTGT |

**References**

1. Schubert S, Heller S, Löffler B, Schäfer I, Seibel M, Villani G, et al. Generation of Rho Zero Cells: Visualization and Quantification of the mtDNA Depletion Process. International journal of molecular sciences. 2015 Apr 30;16(5):9850–65.

2. King MP, Attardi G. Human cells lacking mtDNA: repopulation with exogenous mitochondria by complementation. Science. 1989 Oct 27;246(4929):500 LP – 503.

3. Ludwig C, Günther UL. MetaboLab - advanced NMR data processing and analysis for metabolomics. BMC Bioinformatics. 2011;12(1):366.

4. Goddard Td, Kneller DG. Sparky 3. University of California, San Francisco. San Francisco: University of California;
